# Disruptions, restorations and adaptations to health and nutrition service delivery in multiple states across India over the course of the COVID-19 pandemic in 2020: An observational study

**DOI:** 10.1371/journal.pone.0269674

**Published:** 2022-07-27

**Authors:** Rasmi Avula, Phuong Hong Nguyen, Sattvika Ashok, Sumati Bajaj, Shivani Kachwaha, Anjali Pant, Monika Walia, Anshu Singh, Anshuman Paul, Ayushi Singh, Bharati Kulkarni, Deepak Singhania, Jessica Escobar-Alegria, Little Flower Augustine, Madhulika Khanna, Maitreiyee Krishna, Nandhini Sundaravathanam, Prakash Kumar Nayak, Praveen Kumar Sharma, Prerna Makkar, Puspen Ghosh, Sadhana Subramaniam, Sai Mala, Rakesh Giri, Sameeksha Jain, Santosh Kumar Banjara, Sapna Nair, Sebanti Ghosh, Suman Das, Sumeet Patil, Tanmay Mahapatra, Thomas Forissier, Priya Nanda, Suneeta Krishnan, Purnima Menon

**Affiliations:** 1 International Food Policy Research Institute, Washington, DC, United States of America; 2 Independent Consultant, New Delhi, India; 3 Johns Hopkins University, Baltimore, MD, United States of America; 4 University of Exeter, Exeter, United Kingdom; 5 IDinisght, New Delhi, India; 6 NEERMAN, Mumbai, India; 7 ASER Centre- Pratham Education Foundation, New Delhi, India; 8 Indian Council for Medical Research, New Delhi, India; 9 IIT-Gandhinagar, Gandhinagar, India; 10 FHI 360, New Delhi, India; 11 National Institute of Nutrition, Hyderabad, India; 12 Amazon, Arlington, VA, United States of America; 13 Ashoka University, Sonipat, India; 14 CARE, New Delhi, India; 15 Asian Development Bank, Manila, Philippines; 16 LEAD KREA University, Chennai, India; 17 Bill & Melinda Gates Foundation, New Delhi, India; University of Waterloo, CANADA

## Abstract

**Background:**

Modeling studies estimated severe impacts of potential service delivery disruptions due to COVID-19 pandemic on maternal and child nutrition outcomes. Although anecdotal evidence exists on disruptions, little is known about the actual state of service delivery at scale. We studied disruptions and restorations, challenges and adaptations in health and nutrition service delivery by frontline workers (FLWs) in India during COVID-19 in 2020.

**Methods:**

We conducted phone surveys with 5500 FLWs (among them 3118 Anganwadi Workers) in seven states between August–October 2020, asking about service delivery during April 2020 (T1) and in August-October (T2), and analyzed changes between T1 and T2. We also analyzed health systems administrative data from 704 districts on disruptions and restoration of services between pre-pandemic (December 2019, T0), T1 and T2.

**Results:**

In April 2020 (T1), village centers, fixed day events, child growth monitoring, and immunization were provided by <50% of FLWs in several states. Food supplementation was least disrupted. In T2, center-based services were restored by over a third in most states. Administrative data highlights geographic variability in both disruptions and restorations. Most districts had restored service delivery for pregnant women and children by T2 but had not yet reached T0 levels. Adaptations included home delivery (60 to 96%), coordinating with other FLWs (7 to 49%), and use of phones for counseling (~2 to 65%). Personal fears, long distances, limited personal protective equipment, and antagonistic behavior of beneficiaries were reported challenges.

**Conclusions:**

Services to mothers and children were disrupted during stringent lockdown but restored thereafter, albeit not to pre-pandemic levels. Rapid policy guidance and adaptations by FLWs enabled restoration but little remains known about uptake by client populations. As COVID-19 continues to surge in India, focused attention to ensuring essential services is critical to mitigate these major indirect impacts of the pandemic.

## Introduction

The COVID-19 pandemic has been with the world for over a year now. As the pandemic unfolded, countries took various actions including stringent lockdowns, imposing internal and external travel restrictions, and mandating face masks to stem the spread of the pandemic. Modeling studies, using data from in 118 low-income and middle-income countries (LMICs) suggested disruptions to health and nutrition services would have substantial negative impacts on maternal, child health and nutrition outcomes [1, 2]. For example, under the severe case scenario of coverage reductions of 40 to 52%, and wasting increase of 50%, it would result in 1,157,000 additional child and 56,700 additional maternal deaths over 6 months [1]. In addition, models on the impacts on economies indicated a rise in global poverty [3] and food insecurity [4] especially in LMICs. More recently, a nutrition-focused modeling exercise that combined the impacts of economic slowdowns and health services closures suggested that the world can anticipate substantial increases in maternal and child undernutrition in the coming years [5].

There are indications that nutrition and health service delivery were disrupted around the world, but little primary data is available on the extent and nature of such disruptions. More than 50% countries reported partial disruption to antenatal care (ANC), routine immunization, and sick child services in a key-informant based qualitative study conducted in 105 countries in five WHO regions [6]. In an online global survey, health professionals from LMICs reported decline in availability and utilization of routine services (such as antenatal and postnatal care), and adapting to telemedicine due to transportation restrictions or fear of transmission of COVID-19 [7]. Similar findings of decline in service were documented in a qualitative study conducted in Nepal with a diverse group of community stakeholders [8]. Other studies documented health professionals and frontline workers (FLWs) experiencing high levels of stress and anxiety [7, 9]. Thus, there remains a paucity of published evidence globally and in India on how FLWs are adapting to deliver services to women and children during the pandemic.

India’s lockdown approach to curtailing the spread of COVID-19 resulted in disruption to delivery of maternal and child services at the community level. In the context of India’s malnutrition challenge of 38% stunting [10] and sub-optimal coverage of interventions [11], service disruptions pose great risks to its progress toward stunting reduction, especially in a period where progress appears to have stalled [12]. There was early recognition of the importance of preserving essential services and a first set of policy directives to restart services were released in March and early April 2020 [13]. The early and adaptive policy guidance signaled a strong intent to resume services rapidly (Box 1), but little is known about how this reflected on the ground.

**Box 1. Evolution of policy guidance for delivering health and nutrition services in India during COVID-19**

In India, the COVID-19 pandemic began to unfold in late January and the confirmed cases quickly rose from a few hundred thousand in May to 9.5 million in early December 2020. In late March of 2020, India took stringent lockdown measures to prevent the spread of the COVID-19 pandemic, which were subsequently relaxed. In mid-April, the Ministry of Health and Family Welfare issued comprehensive guidance to continue provision of essential health and nutrition services. The guidance was updated periodically, and states across India adapted it to their context or issued their own guidance.

We assessed state policy guidance from March until September 2020 among the seven states (Bihar, Chhattisgarh, Madhya Pradesh, Odisha, Tamil Nadu, Telangana, and Uttar Pradesh), and compared it with the national guidance. We found the guidance varied by state and by the type of service (S1 Fig).

Village centers remained closed in all states, affecting some key services such as hot-cooked meals and growth monitoring. However, for fixed-day events (Village Health and Nutrition Days, VHND) for all beneficiaries, only Odisha issued early guidance, which included instructions to frontline workers on staggering beneficiary groups during the day and maintaining physical distance. Bihar and Uttar Pradesh issued orders in April to hold VHNDs only in areas with risk of low rate of infection and only for a few beneficiaries at any given time. These orders pre-dated the national guidance, which was issued in May.

For home visits and counselling, states provided local contextually relevant guidance, in alignment with national guidance on counselling during home visits. Only Bihar, Madhya Pradesh, Odisha and Uttar Pradesh provided early guidance on counselling and with an intent to reach all beneficiaries with life-stage specific counselling. Madhya Pradesh government encouraged FLWs to use their phones and instant messaging features to share information and counsel groups of beneficiaries.

Early policy guidance (March 2020) was available for food supplementation across all five states, directing FLWs to deliver food supplements to beneficiaries’ homes, although there was no known national level directive at that time. For iron and folic acid (IFA) supplementation and immunization, states followed national guidance, except Odisha, which continued to provide immunization in community settings. For growth monitoring, Odisha and Madhya Pradesh had guidance in place prior to the national guidance. Overall, state guidance on immunization services was in alignment with the national guidance, which included service delivery at health facilities and at restricted community sessions. Odisha’s guidance pre-dates national guidance and included provision of immunization on designated days in the community.

Together, the national and state policy guidance enabled service restorations. The policy guidance evolved rapidly, responding to the evolving COVID-19 situation. While for most services, states followed the national guidance, states were also not fully dependent on them and contextualized it to their local needs. 

India drew on its strong cadre of nearly 2.42 million FLWs including Anganwadi Workers (AWWs) (Box 1), Accredited Social Health Activists (ASHAs), and Auxiliary Nurse Midwives (ANMs). Anecdotal insights had emerged early in the pandemic that FLWs were delivering essential products to beneficiary homes and using phones to provide counselling for beneficiaries when services were disrupted, while navigating challenges including personal and beneficiary anxieties related to the pandemic [14]. However, even a year into the pandemic, empirical evidence on service disruptions as well as restorations, adaptations and challenges to service delivery during the pandemic is severely limited, particularly from resource-poor settings like India. Therefore, together with a network of national researchers in India, we undertook a study to: (1) Characterize the extent of disruptions to and restorations of health and nutrition service delivery; (2) Examine geographic and temporal variability in service disruptions and restorations; and (3) Understand FLW adaptations that enabled the restoration of the delivery of routine health and nutrition services.

## Methods

### Context

To understand how FLWs responded to the government guidance and delivered essential interventions during the pandemic, we built a multi-partner consortium with six research organizations (ASER Centre- Pratham Education Foundation, CARE India/Oxford Policy Management Ltd, Evidence for Policy Design India at Krea University [EPoD India], IFMR LEAD KREA, International Food Policy Research Institute [IFPRI] and the Indian Council for Medical Research-National Institute of Nutrition [NIN]). The multi-partner collaboration process has been described previously [15].

We drew on two primary data sources: (1) cross-sectional phone surveys with FLWs and (2) publicly available administrative data from the Health Management Information System (HMIS).

### Multi-state phone survey

**Fig 1:**
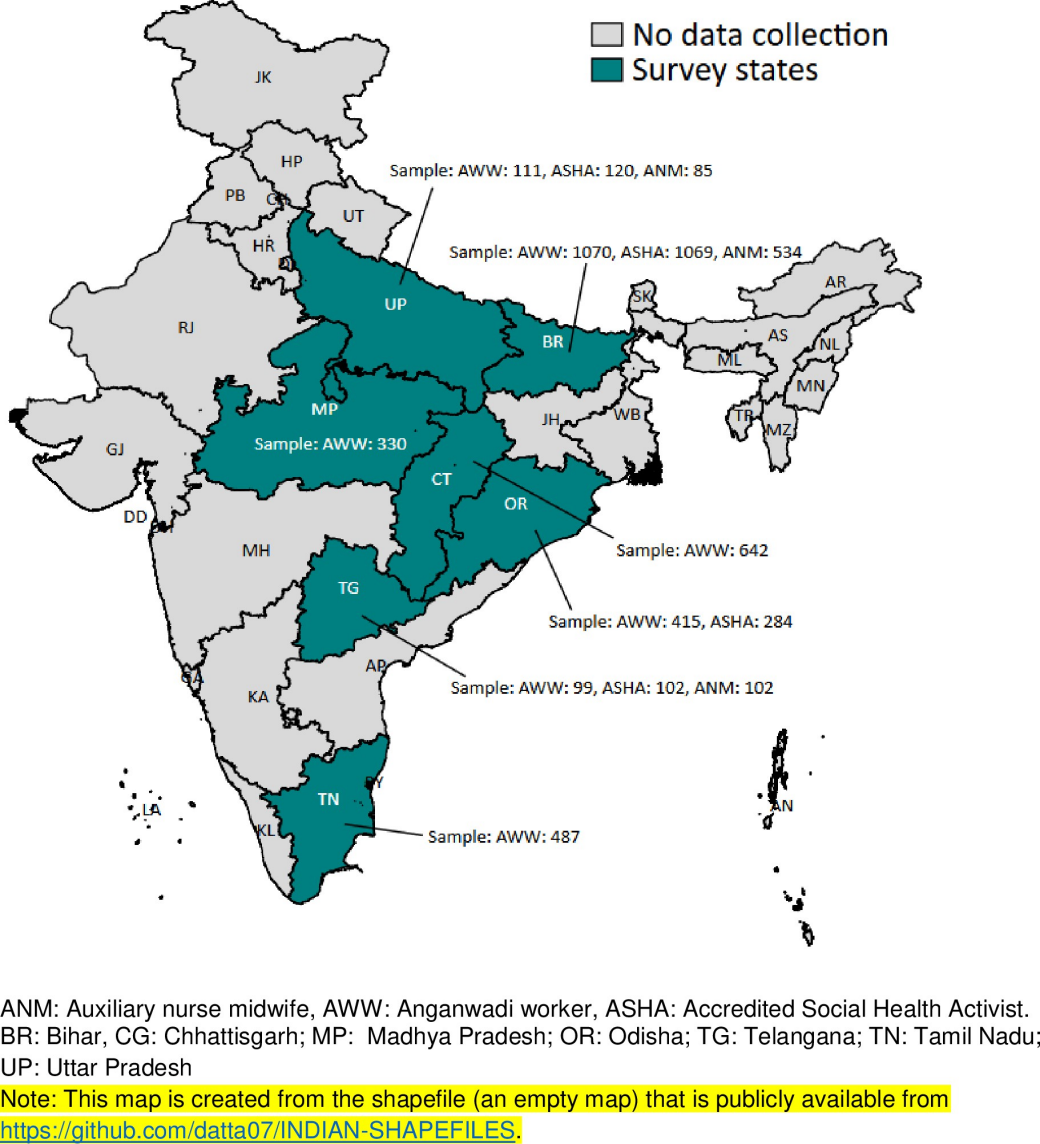
Phone survey in India: State and sample size. ANM: Auxiliary Nurse Midwife, AWW: Anganwadi Worker, ASHA: Accredited Social Health Activist. BR: Bihar, CG: Chhattisgarh; MP: Madhya Pradesh; OR: Odisha; TG: Telangana; TN: Tamil Nadu; UP: Uttar Pradesh.

We conducted phone-based interviews with 5500 FLWs in 71 districts from seven states (Bihar, Chhattisgarh, Madya Pradesh, Odisha, Tamil Nadu, Telangana, and Uttar Pradesh) between August and October 2020. The sampling strategy and sample size were opportunistic and were determined based on existing data collection opportunities within ongoing studies or existing research collaborations with state governments. For example, the phone survey in Uttar Pradesh was based on pre-existing contacts from the main impact evaluation of maternal nutrition managed by IFPRI in collaboration with Alive & Thrive. In some states (Bihar, Telangana, and Uttar Pradesh), we collected data from all three types of FLWs while in others, we collected data only from AWWs. Detailed sample sizes for each type of FLW surveyed in each state are shown in Fig 1.

The data collection questionnaire (S1 File) drew on a framework of indicators related to India’s National Nutrition Mission [16]. Questions on service delivery, challenges and adaptations covered facility- and outreach-based interventions. FLWs were asked to recall services they had delivered in April (during the COVID-19 lockdown) and in July-September (the one month preceding the survey), adaptations to deal with challenges, training and incentives received, COVID-19 related duties, and effect of COVID-19 on their households.

### Analysis of administrative data from health systems

We used HMIS data from December 2019 to June 2020, which provide month-wise information on provision and utilization of health services at national and sub-national (state and district) levels [17]. We selected four indicators from the HMIS that aligned with those in the phone survey: 1) Number of village centers / urban primary health centers that conducted Village Health and Nutrition Days (VHNDs); 2) Number of pregnant women who received ≥4 ANC check-ups; 3) Number of pregnant women given 180 iron and folic acid (IFA) tablets; and 4) Number of children aged 9–11 months who received full immunization (BCG, 3 doses of DPT, 3 doses of OPV and measles) [18]. The administrative data complements the phone surveys because it captures service provision and utilization before and during the pandemic and provides insights on 704 of 733 districts in India. Data from the months after June 2020 were unavailable in the public domain at the time of the analysis.

#### Statistical analysis

We define three time periods to analyze disruptions and restorations in service delivery:

- T0: The pre- COVID-19 period (December 2019, for which only administrative data are available)
- T1: The lockdown period (April 2020, stringent lockdown time)
- T2: The post-lockdown period (July to September 2020 for phone surveys; June 2020 for administrative data).

*Using the phone survey*, we compared the changes in service provision between T1 (lockdown) and T2 (post-lock down). We summarize the challenges faced in each state and the types of adaptations made by FLWs to support service delivery. Although we interviewed all three cadres of FLWs, we present findings only on AWW (n = 3118) because they were interviewed in all geographies. Findings from interviews with other FLWs were similar (not shown).

*Using the administrative data*, we estimated disruptions and restorations by calculating the percentage of change in service delivery at three time points: between T0 and T1 (service disruption); between T1 and T2 (service restoration); and finally, between T0 and T2 (service restoration compared to the pre-pandemic period).

Change was estimated as:

% change between T0 and T1 = ((service in T1- service in T0)/service in T0) *100.

All statistical analysis was undertaken using Stata version 16.

### Ethical approval

This study received ethical approval from the Institutional Review Board at the International Food Policy Research Institute (IRB# 00007490). Permissions for data collection were also obtained from state governments. Oral consent was obtained from the study participants at the beginning of the phone survey. The administrative data are publicly available at aggregate level with no identifiers and require no additional ethical or administrative approval for their use.

## Results

### Characteristics of FLWs

Response rate varied by FLW cadre and state (S1 Table). On average, AWWs were 39–45 years old and had been working for 13–17 years. In Bihar, Madhya Pradesh, Tamil Nadu, and Telangana more than 90% of AWWs had phone access, whereas 75% in Chhattisgarh, 20% in Uttar Pradesh, and <1% in Odisha had access to smart phones (S1 Table).

### Delivery of services during the lockdown (T1, April 2020) and after the lockdown (T2, July-September 2020) based on phone survey

**Fig 2:**
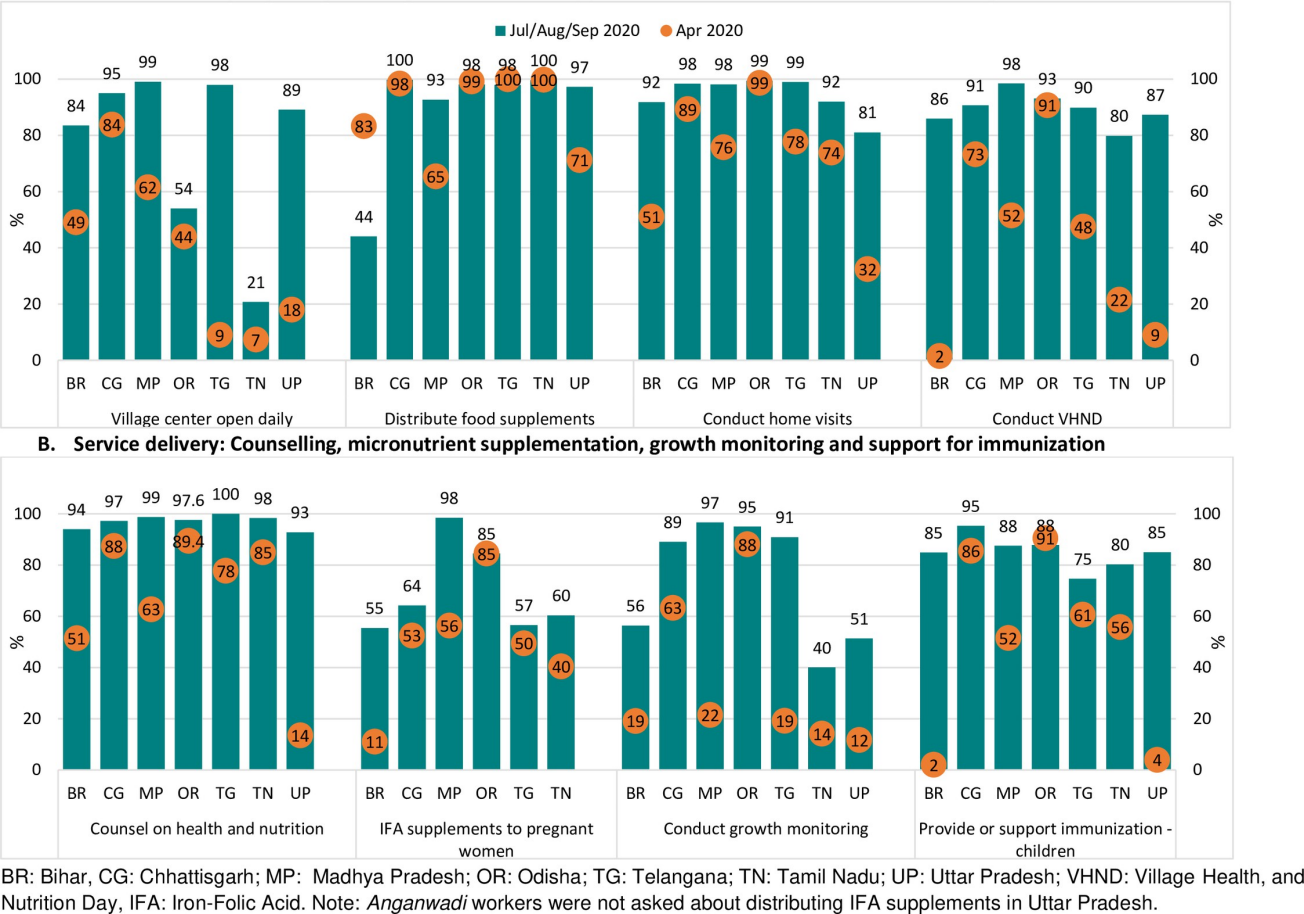
*Anganwadi* worker service delivery during the COVID lockdown in April 2020 and in July/August/September 2020, by state. A. Service delivery: Opening of village center, food supplementation, home visits, and conducting VHND. B. Service delivery: Counselling, micronutrient supplementation, growth monitoring and support for immunization. BR: Bihar, CG: Chhattisgarh; MP: Madhya Pradesh; OR: Odisha; TG: Telangana; TN: Tamil Nadu; UP: Uttar Pradesh; VHND: Village Health, and Nutrition Day, IFA: Iron-Folic Acid. Note: *Anganwadi* workers were not asked about distributing IFA supplements in Uttar Pradesh.

The village centers, which are community-level centers for delivering maternal and child services, were not opened daily in most states in April 2020 (T1), except in Chhattisgarh. In T2, most AWWs across states reported opening the centers except in Tamil Nadu (21%) and Odisha (54%) (Fig 2).

Most AWWs (65 to 100%) reported distributing food supplements to beneficiaries in T1 and nearly all of them, across all states, reported service resumption in T2 (Fig 2). Reports of holding fixed day services such as VHNDs varied widely across states, from as low as 2% in Bihar or 9% in Uttar Pradesh to as high as 73–91% in Chhattisgarh and Odisha. In T2, over 80% of AWWs reported holding a VHND.

Most AWWs conducted home visits (74 to 99%) even during the lockdown (T1) except in Bihar (51%) and Uttar Pradesh (32%). In T2, however, 92% and 81% AWWs in Bihar and Uttar Pradesh respectively, reported conducting home visits. Barring Uttar Pradesh (14%), more than 50% of AWWs reported providing counseling on health and nutrition in T1 and nearly all AWWs across states reported doing so in T2. Between 11–85% AWWs reported providing IFA supplements to pregnant women in April in most states. In T2, majority of AWWs (55–98%) across states reported providing IFA.

A majority of AWWs in Chhattisgarh (63%) and Odisha (88%) reported conducting growth monitoring for children in T1, while in the other states only some AWWs (12–22%) reported doing so. In T2, majority of AWWs in Chhattisgarh, Madhya Pradesh, Odisha, and Telangana (89–97%) reported conducting growth monitoring; but fewer AWWs (40–56%) reported it in other states. More than 50% of AWWs in most states in T1 (except Bihar and Uttar Pradesh) and in all states in T2 reported supporting immunization services (Fig 2). In addition to delivering nutrition services, AWWs performed several COVID–19 specific duties including identifying households with illness, collecting information on visitors or migrants, providing information on COVID-19, and managing quarantine centers.

### Delivery of services before the pandemic (T0, December 2019), during the lockdown (T1, April 2020), and after the lockdown (T2, June 2020) based on health administrative data

**Fig 3:**
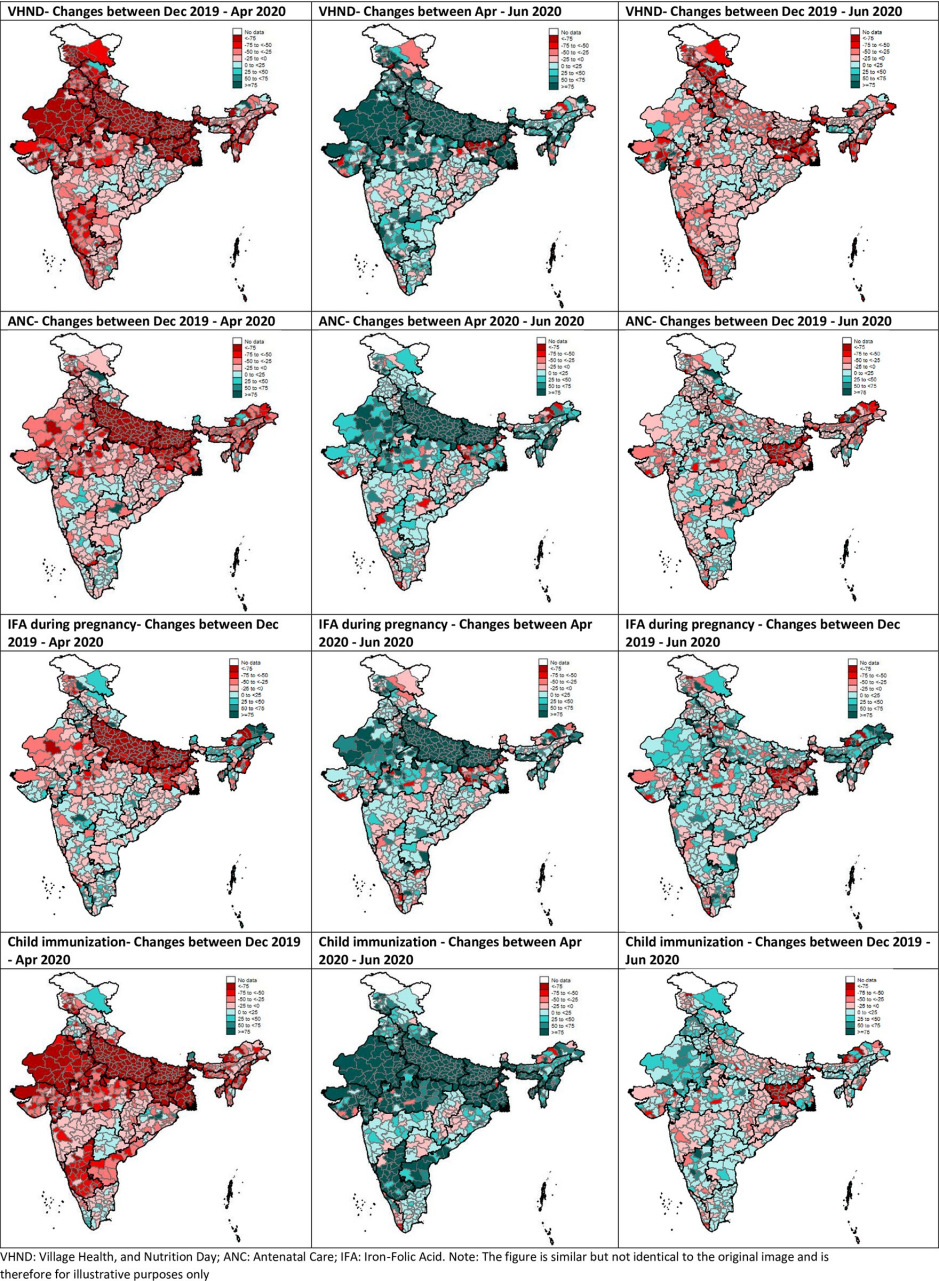
Services disruption and restoration during the COVID pandemic: Results from health management information system data. VHND: Village Health, and Nutrition Day; ANC: Antenatal Care; IFA: Iron-Folic Acid.

Fig 3 shows disruption and restoration of services during the pandemic based on the *administrative (HMIS) data*. Between T0 and T1, on average conducting of VHND was reduced by 53%. Disruptions to conducting VHND were observed in 653 of 698 districts (Fig 3). In 288 of the 653 districts, there was >50% reduction in conducting VHND in T1. Service disruption varied geographically; Bihar and Uttar Pradesh, two of India’s largest states, were severely affected. VHNDs resumed in over 80% of districts after the lockdown was lifted but remained lower than in T0.

Between T0 and T1, utilization of ANC, IFA during pregnancy, and immunization were reduced by 33%, 16%, and 47%, respectively. Higher than 50% disruption was noted in 31%, 24%, and 48% of districts for ANC, IFA during pregnancy, and immunization, respectively. Overall, disruptions were observed in 84% of districts for ANC, 67% districts for IFA supplements and 88% districts for immunization.

Uttar Pradesh had greatest disruption, followed by Bihar and Madhya Pradesh. Most districts had restored service delivery for pregnant women and children by T2, but levels had not yet reached T0 levels yet, suggesting lingering disruptions.

### Adaptations to service delivery during the pandemic

**Fig 4:**
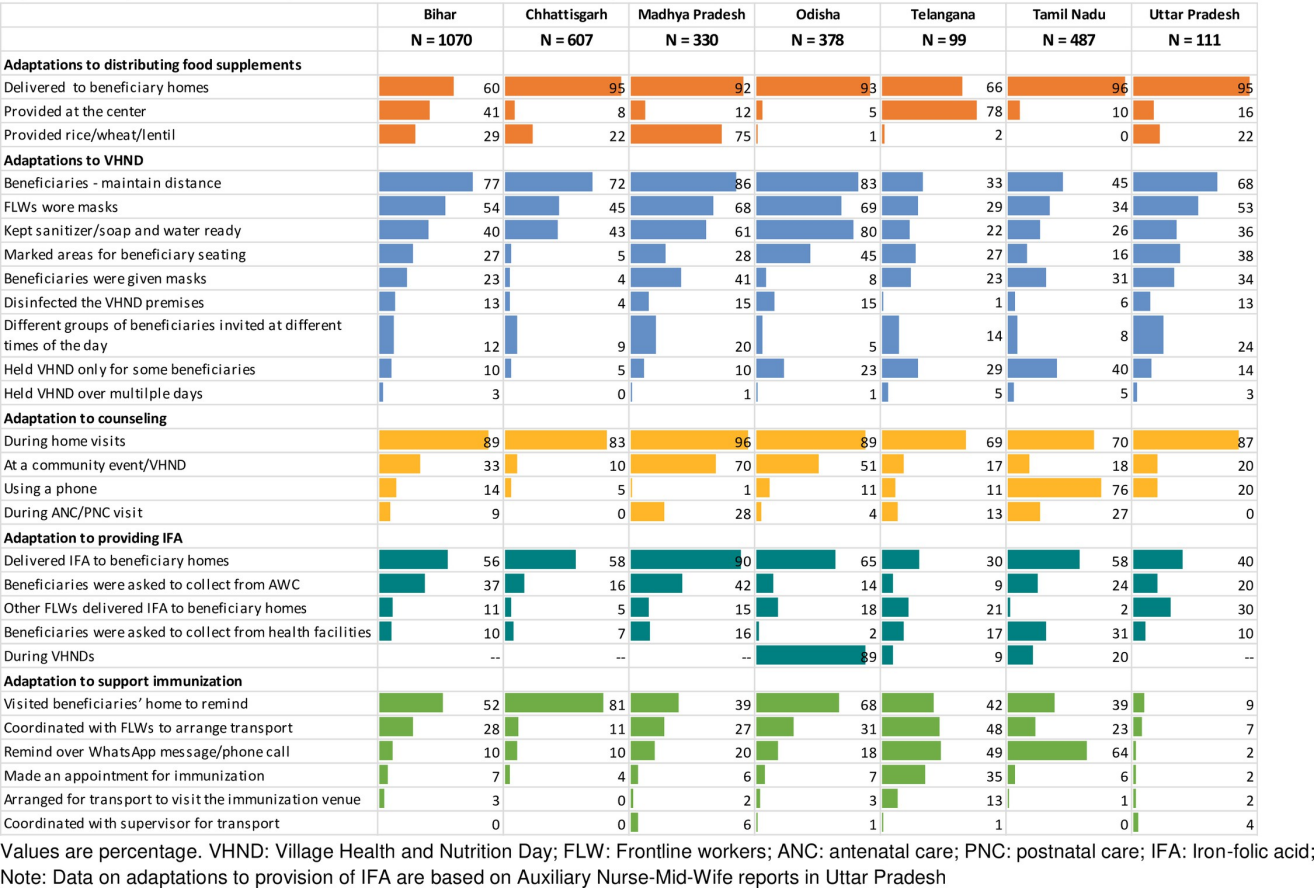
Adaptations made to provide services during COVID-19 pandemic (April 2020). VHND: Village Health and Nutrition Day; FLW: Frontline workers; ANC: antenatal care; PNC: postnatal care; IFA: Iron-folic acid. Note: Data on adaptations to provision of IFA are based on Auxiliary Nurse-Mid-Wife reports in Uttar Pradesh.

AWWs made several adaptations to preserve service delivery during T1 and T2, including providing services at beneficiary homes, ensuring physical distancing when providing center-based services, coordinating with other FLWs to arrange for visits to health centers for ANC for pregnant women or arrange for visits for immunization, and using phones to deliver information (Fig 4). Between 60 to 96% AWWs home delivered food supplements, except in Telangana, where 78% AWWs reported providing food supplements at the center. Most AWWs provided health and nutrition counselling (69–96%), IFA supplements (30–90%), and reminded beneficiaries of immunization services (9–81%) during home visits.

To hold VHNDs, 33 to 86% AWWs reported asking beneficiaries to maintain distance, 29 to 69% wore masks and 22 to 80% provided sanitizer/soap and water on the VHND premises. Finally, phones were used to adapt service delivery and supervision. Most AWWs in Tamil Nadu used phones for counseling or contacting beneficiaries to remind them about immunization (Fig 4). Most AWWs across all states also used phones to communicate with their supervisors in T1 and continued to do so during T2, although in-person communication increased in T2 (S2 Fig). Majority of AWWs demonstrated knowledge of measures for protection against COVID-19 (S2 Table).

### Challenges in delivering services during the pandemic

**Fig 5:**
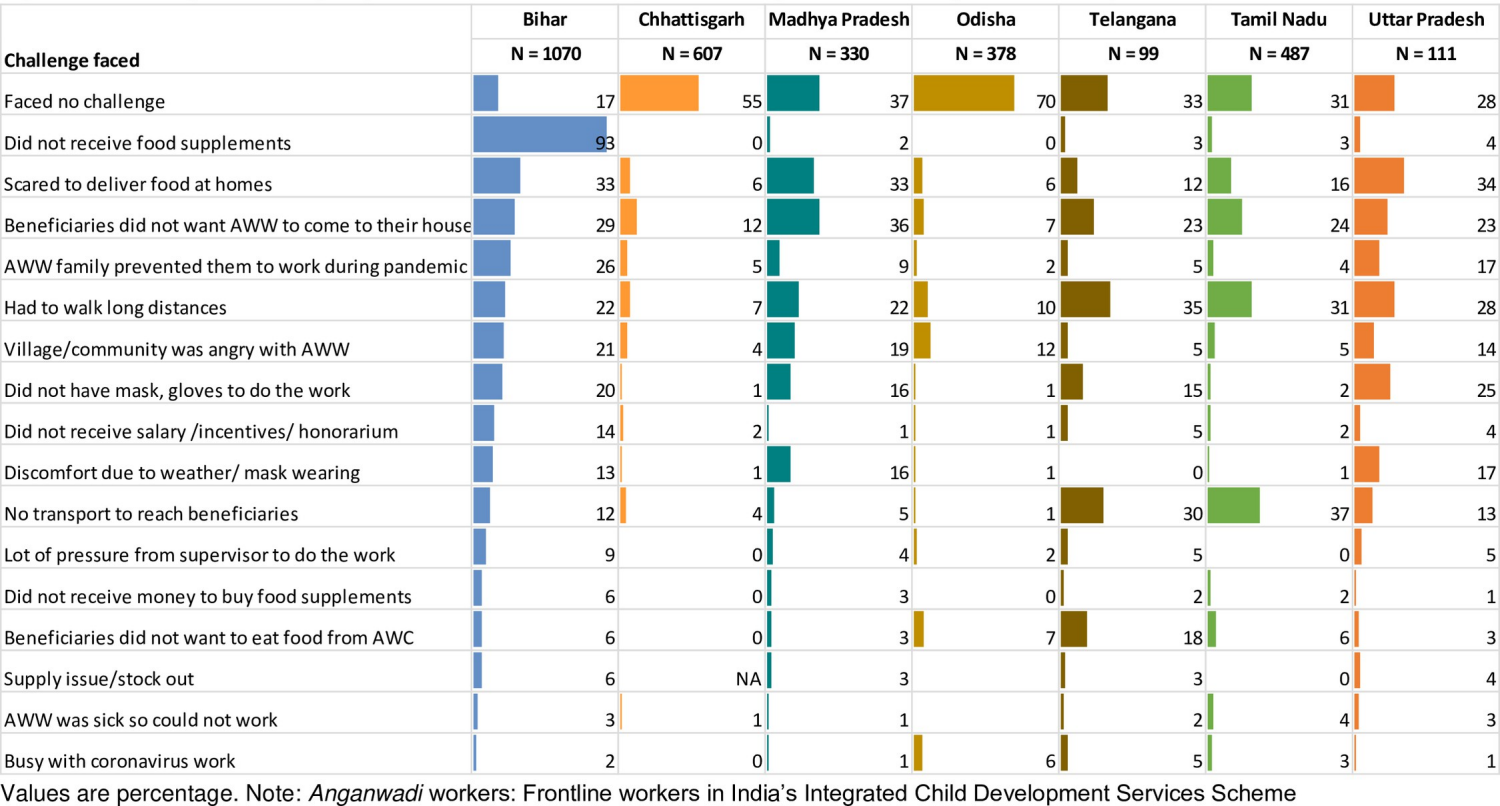
Challenges faced by *Anganwadi* workers (AWWs) in delivering services during COVID-19 (April 2020). Note: *Anganwadi* Workers: Frontline workers in India’s Integrated Child Development Services Scheme.

Challenges faced in delivering services varied across states (Fig 5). Nearly a third of AWWs cited walking long distances and lack of transport as challenges. Except in Chhattisgarh (12%) and Odisha (7%), some AWWs in other states (between 23 and 36%) reported beneficiaries’ resistance to home visits. Some AWWs (12–21%) in some states also reported that communities were angry with them, while almost a third in Bihar, Madhya Pradesh, and Uttar Pradesh also reported being scared to home deliver food supplements.

#### Resources and FLW training to respond to COVID-19

Reported receipt of personal protective equipment (PPE) varied widely; 28–90% AWWs reported receiving masks, 5–62% gloves, and 7–70% sanitizer/soap. More than half the AWWs across states reported receiving training on COVID–19 symptoms, protecting oneself, and communicating with the community (S2 Fig).

## Discussion

This study draws on two data sources to assess disruptions, restorations and adaptations to health and nutrition service delivery in multiple states across India during COVID-19. Majority of AWWs reported low service provision including opening of village centers, holding of VHND, immunization and growth monitoring during the lockdown. These services, however, were restored after the lockdown with some adaptations. Health system administrative data supported findings of widespread service disruptions across India in April 2020. In June 2020, most services had begun to be restored but were below the pre-pandemic coverage levels. FLWs used a variety of adaptive mechanisms to deliver services, but also noted facing challenges such as fear of visiting homes, beneficiaries’ resistance to FLWs, and transportation difficulties.

Center-based services such as VHND and immunization were most disrupted, whereas services that were amenable to delivery via home visits or using technology were less likely to be disrupted. After the country-wide stringent lockdown was relaxed, state-specific guidelines on service delivery were implemented based on the differences in the COVID-19 case scenarios. The centers were not opened for routine service delivery across states given the pandemic situation. These disruptions are aligned with global findings where almost every country reported some extent of service disruptions. Among the 105 countries in the five WHO regions, 80% had essential healthcare packages in place prior to the pandemic and 66% of all countries had identified a coreset of services to continue during the pandemic. However, 56% of all countries reported disruptions to ANC and 61% to immunization services [6], both of which are center-based services in most countries.

Findings from the administrative data bolster the insights on service disruptions reported by the AWWs in the phone survey. Disruptions were large and extended across the country, especially in states such as Uttar Pradesh, Bihar and Madhya Pradesh. With an annual birth cohort of nearly 8.3 million (35·9% of all babies born in India) in these states [19], the disruptions in these large states would likely have a disproportionate impact on health outcomes as has been observed with disproportionate burden of stunting prevalence [20]. Although the findings on restorations are promising, the comparison with the pre-COVID timeframe indicates that full restoration remains a challenge. This has implications for India’s progress towards meeting nutrition goals, especially in states where pre-pandemic challenges were already substantial [21]. Continued analyses of administrative data can help assess the pace and extent of the restoration. By end of 2020, service delivery related to IFA supplementation was restored at the pre-pandemic level, as shown in HMIS dataset available on the dashboard tracking anemia prevention program in India [22]. At the time of writing this paper, administrative data after June 2020 remains unavailable in the public domain.

Across all states, food supplementation was the least affected service in our study, likely because these were dry commodities available at village centers and could be home delivered with minimal contact. In addition, under the National Food Security Act [23], the government is mandated by the law to ensure food supplementation for women and children in the first 1,000 days; therefore, it is likely, policy guidance was issued for door-step delivery of food commodities. In the context of rising food insecurity due to COVID-19 [4, 24], lack of access to nutritious, safe, and affordable diets could exacerbate maternal and child malnutrition [7, 25, 26]. Such food supplementation programs could act as a safeguard against dire outcomes in the interim, at least partially, while efforts are made to strengthen the food systems to deliver for nutrition.

In our study, FLWs used a variety of approaches to adapt service delivery in ways that ensured reach of at least some key services. For example, food and micronutrient supplements, which are usually provided at centers, were home delivered in several states, while a directive was issued to provide direct benefit transfer to the beneficiary bank account instead of providing food supplements in Bihar. When services such as VHNDs were conducted in-person at the centers, measures were taken to stagger beneficiary visits, maintain physical distance, wear masks, and ensure hygiene. Similar precautions and adaptations were reported from across the globe such as social distancing inside the clinics, instituting additional cleanliness measures, spacing appointments [7], using telemedicine [7, 25], adjusting supply chain or ways of dispensing medicines, redirecting patients to alternate health care facilities, digital health and intensified communication [6].

Counseling, which is usually provided at the center or during home visits, was provided over phones, especially where most FLWs had mobile phones. These findings are promising given the evidence for mobile technology-based interventions in improving delivery of maternal and child health services [27–29] as well as health practices. As mobile phone access increases both among FLWs and beneficiary populations, technology could have great potential to strengthen delivery, particularly during challenging times. However, without clear guidelines for care provision, improvement of infrastructure and technological literacy, and addressing financial barriers, inequities in access to healthcare are likely to become exacerbated [30].

Policy guidance plays an important role in signaling priorities for service delivery in India. In this case, early policy guidance in March 2020 prioritized reinstating of services through reimagining delivery modalities for food supplementation. This was followed by guidance in April 2020 for ANC, immunization, IFA supplementation, and much later for vitamin A supplementation. In addition to national policy prioritization, however, there was state-level variability both in issuance of state-level guidance and implementation of that guidance (Box 1) [31]. The pace and extent of state guidance could reflect pre-existing state-level policy prioritization of health and nutrition and state-level implementation capabilities. It is plausible that states such as Chhattisgarh and Odisha which had strong pre-pandemic policy support for nutrition and strong delivery systems [32, 33] were able to respond relatively quickly to restore services. Global data also indicate that disruptions were greater in LMICs compared to upper-middle and high-income countries [6], which could reflect differences in the strength of their health systems.

Globally, FLWs are well-recognized to be central to health systems in LMICs and play a major role in improving maternal and child health outcomes [34]. Our study indicates that FLWs were indeed continuing to deliver services despite logistical challenges, personal fears of contracting the virus, social challenges of being feared and shunned by the community, and uneven receipt of protective equipment. Pre-pandemic, India’s FLWs were already operating within a complex milieu of personal, community, and organizational factors and managing multiple challenges with limited support [35]. In the context of the challenges imposed by COVID-19, where FLWs are performing additional COVID-19 duties, it is imperative that they receive the necessary support including PPE, psychosocial support, travel and other allowances [36]. Particularly, it is important to recognize that the pandemic has affected AWWs’ lives as well and proving them a supportive environment is critical to their motivation (**Box** 2). As home visits are an important platform for service delivery, it is equally important to raise community awareness of FLW duties during the pandemic to ensure FLWs’ safety and acceptance.

**Box 2. Impact of COVID-19 on the lives of *Anganwadi* Workers (AWWs) in seven states in India**

India’s policy framework for nutrition covers most of the evidence-based interventions in the first 1000 days. These interventions are delivered through its two large-scale national programs–the Integrated Child Development Services (ICDS) and the National Health Mission (NHM) by three cadres of frontline workers—Anganwadi Workers (AWWs, 1.3 million), Accredited Social Health Activists (ASHAs, 0.92 million), and Auxiliary Nurse Midwives (ANMs, 0.2 million). Most interventions are delivered at the community level at the *Anganwadi* centers and during home visits. AWWs provide services to adolescent girls, pregnant and lactating mothers, and children aged between 0 to 6 years; the services include food supplementation, health and nutrition counseling, growth monitoring and referral services, support immunization, health check-ups, and pre-school education. ASHAs are recruited one per 1000 population to raise awareness about health and its social determinants and promote existing health services and facilitate their utilization. ANMs are present at the sub-center level and provide maternal and child health care services. In 2016, at the national level, the highest level of coverage for key nutrition interventions was about 65%, and for most it was approximately 50% or less, indicating sub-optimal coverage.

During the pandemic, AWWs continued to deliver essential services, adapting to the conditions imposed by the pandemic. In addition, they conducted surveys to identify households with illness, collected information on visitors or migrants, provided information on COVID-19, and managed quarantine centers (S2 Fig).

COVID-19 upended lives of millions of people, disrupting livelihoods, economy, food security, and health care services; the personal lives of AWWs in India were no exception. The types of challenges faced by AWWs varied by state (S3 Table).

- 37–65% of AWW households were affected by unemployment or loss of income
- 15–54% of AWWs faced challenges in procuring food
- 28–47% of AWWs were affected by shop closures
- 21–32% of AWW households faced difficulties due to long distance travel restrictions
- Fear of family members getting sick, burden of additional home duties, and traveling long distance for work were some other challenges faced by some AWWs in some states.

Despite many personal challenges, the AWWs persevered and continued to provide services putting themselves and their families at risk. Similar challenges were reported by other cadres of frontline workers. It is seldom that these workers are treated with the dignity, respect, and remuneration they deserve.

Some limitations of the study are worth mentioning. Our study was conducted during the pandemic and therefore the assessment of service provision was conducted using phone interviews. There is potential for desirability bias from self-reported data and over-reporting of service delivery, and this could have been further amplified because of phone-based interviews [37]. Data on service provision during the lockdown (April 2020) was collected retrospectively (after 3–5 months) which could have potential recall bias. The primary data collection did not use uniform or representative sampling frames across states, thus limiting generalizability or assessment of differences. However, the similarity of findings across the phone surveys and the administrative data lend credibility to the survey results and enhance generalizability. This similarity also suggests that if countries are unable to restore routine monitoring information systems during an emergency, rapid phone-based surveys of providers could be an efficient means to obtaining information on the status of service delivery. Finally, household surveys are essential to understand the full extent of the impact of disruptions and restorations on the beneficiary-level coverage of these services. Although FLWs report delivering services, it is likely that not all beneficiaries are utilizing them due to travel restrictions or fear of contracting COVID-19 [38].

## Conclusion

Our study, built on a national multi-partner collaboration, has generated data from phone-based surveys across several states in India. Complemented by an analysis of administrative data, it provides insights on service disruptions, restorations, challenges, and adaptations across India. A key feature of our study is the collective effort of different organizations to invest in large-scale data collection using remote survey techniques during the pandemic. The findings on restorations and adaptations are promising; however, they are indicative of how both pre-existing inequalities and capabilities across states have had implications for adapting service delivery during a crisis.

India’s strong cadre of health and nutrition FLWs has played a tremendous role in service restorations in difficult times, and the pandemic has revealed, yet again, the importance of supporting these FLWs across India. In the context of the ongoing COVID-19 surge in India [39], and the strong likelihood of subsequent waves, there remains an urgent need to reduce gaps in training, PPE, and incentives to help FLWs safely restore of routine services. Given that little is known about the impacts of service disruptions or restorations on coverage and equity of services, further insights are also needed from household data. Finally, the promising role of technology as an option for delivering counseling services should continue to be studied.

## Supporting information

S1 Table Characteristics of frontline workers delivering health and nutrition services. Anganwadi worker, Accredited Social Health Activist, and Auxiliary Nurse Midwife are three cadres of frontline workers who are part of India’s national programs that deliver maternal and child nutrition services. “—” No data available. Notes: The exceptionally high response rates in Bihar were because CARE-India has ongoing learning assessments in the state and therefore have established field teams with contacts with FLWs for the past five years. In Chhattisgarh and Tamil Nadu, FLW phone numbers were obtained from the government and most numbers were valid. In Telangana, NIN had prior relationship with ASHAs who were interviewed first, and the trust building helped the team in interviewing AWWs whose phone numbers were obtained from ASHAs. In Madhya Pradesh and Uttar Pradesh, FLWs from studies conducted in 2018 and 2019, respectively, were interviewed and hence there were more unreachable phones, wrong numbers, and refusal rates. The analytic sample includes 4,293 FLWs (Fig 1). As AWWs were the common cadre interviewed in all the states, we present results of AWW surveys; the findings were similar to that of the other cadres (Web appendix).
(DOCX)

S2 Table Knowledge of frontline workers. (DOCX)

S3 Table *Anganwadi workers* affected by COVID-19. (DOCX)

S1 Fig State level policy guidance for delivering health and nutrition services during COVID-19. (TIF)

S2 Fig Resources for and responsibilities of *Anganwadi* workers to respond to COVID-19. (TIF)

S1 File Questionnaire. (DOCX)

## References

[1] Roberton T, Carter ED, Chou VB, Stegmuller AR, Jackson BD, Tam Y, et al. Early estimates of the indirect effects of the COVID-19 pandemic on maternal and child mortality in low-income and middle-income countries: a modelling study. Lancet Glob Heal. 2020;8(7):e901–8. 10.1016/S2214-109X(20)30229-1 PMC7217645 32405459

[2] Headey D, Heidkamp R, Osendarp S, Ruel M, Scott N, Black R, et al. Impacts of COVID-19 on childhood malnutrition and nutrition-related mortality. Lancet. 2020 Aug 22;396(10250):519–21. doi: 10.1016/S0140-6736(20)31647-0 32730743 PMC7384798

[3] Laborde D, Martin W, Vos R. Impacts of COVID‐19 on global poverty, food security, and diets: Insights from global model scenario analysis. Agric Econ. 2021 May 8;52(3):375–90. doi: 10.1111/agec.12624 34230728 PMC8251321

[4] Laborde D, Martin W, Swinnen J, Vos R. COVID-19 risks to global food security. Science. 2020 Jul 31;369(6503):500–2. doi: 10.1126/science.abc4765 32732407

[5] Osendarp S, Akuoku JK, Black RE, Headey D, Ruel M, Scott N, et al. The COVID-19 crisis will exacerbate maternal and child undernutrition and child mortality in low- and middle-income countries. Nat Food. 2021 Jul 19;2(7):476–84. 10.1038/s43016-021-00319-4 37117686

[6] World Health Organization. Interim report 27 August 2020. Pulse survey on continuity of essential health services during the COVID-19 pandemic. 2020. Available from: https://www.who.int/publications/i/item/WHO-2019-nCoV-EHS_continuity-survey-2020.1

[7] Semaan A, Audet C, Huysmans E, Afolabi B, Assarag B, Banke-Thomas A, et al. Voices from the frontline: findings from a thematic analysis of a rapid online global survey of maternal and newborn health professionals facing the COVID-19 pandemic. BMJ Glob Heal. 2020 Jun;5(6). doi: 10.1136/bmjgh-2020-002967 32586891 PMC7335688

[8] Singh DR, Sunuwar DR, Shah SK, Karki K, Sah LK, Adhikari B, et al. Impact of COVID-19 on health services utilization in Province-2 of Nepal: a qualitative study among community members and stakeholders. BMC Health Serv Res. 2021 Feb 24;21(1):174. doi: 10.1186/s12913-021-06176-y 33627115 PMC7903406

[9] Cabarkapa S, Nadjidai SE, Murgier J, Ng CH. The psychological impact of COVID-19 and other viral epidemics on frontline healthcare workers and ways to address it: A rapid systematic review. Brain, Behav Immun—Heal. 2020 Oct;8(June):100144. doi: 10.1016/j.bbih.2020.100144 32959031 PMC7494453

[10] International Institute for Population Sciences (IIPS), ICF. National Family Health Survey (NFHS-4), 2015–16: India. Mumbai; 2017.

[11] Chakrabarti S, Raghunathan K, Alderman H, Menon P, Nguyen P. India’s Integrated Child Development Services programme; equity and extent of coverage in 2006 and 2016. Bull World Health Organ. 2019 Apr 1;97(4):270–82. f doi: 10.2471/BLT.18.221135 30940984 PMC6438246

[12] Chatterjee P. India’s child malnutrition story worsens. Lancet Child Adolesc Heal. 2021 May;5(5):319–20.

[13] Ministry of Health and Family welfare. Enabling Delivery of Essential Health Services during the COVID 19 Outbreak: Guidance note. 2020;22(2017):60–70. Available from: https://www.mohfw.gov.in/pdf/EssentialservicesduringCOVID19updated0411201.pdf

[14] Nair S, Avula R, Ashok S, Ramu K. The Role of Last Mile Community Workers in a Pandemic: A Case Story from Tamil Nadu. IFPRI South Asia. 2020. Available from: https://southasia.ifpri.info/2020/06/08/the-role-of-last-mile-community-workers-in-a-pandemic-a-case-story-from-tamil-nadu/

[15] Avula R, Nguyen P, Ashok S, Bajaj S, Kachwaha S, Pant A, et al. Building a multi-partner collaboration for developing a learning agenda on frontline workers during COVID-19: A case study from India. J Epidemiol Community Med. (Under review)

[16] Menon P, Avula R, Sarswat E, Mani S, Jangid M, Singh A, et al. Tracking India’s Progress on Addressing Malnutrition: What will it take?. New Delhi; 2020. Available from: https://ebrary.ifpri.org/utils/getfile/collection/p15738coll2/id/134227/filename/134438.pdf

[17] National Health Systems Resource Centre. Health Monitoring Information System.

[18] Aayog NITI, Bank World, Ministry of Health and Family Welfare. Healthy States Progressive India: Report of the Ranks of the States and Union Territories. 2019. Available from: http://social.niti.gov.in/uploads/sample/health_index_report.pdf

[19] Ministry of Home Affairs. Vital Statistics of India based on the Civil Registration System 2018. 2018.

[20] Menon P, Headey D, Avula R, Nguyen PH. Understanding the geographical burden of stunting in India: A regression-decomposition analysis of district-level data from 2015–16. Matern Child Nutr. 2018;14(4):e12620. doi: 10.1111/mcn.12620 29797455 PMC6175441

[21] India State-Level Disease Burden Initiative Malnutrition Collaborators. The burden of child and maternal malnutrition and trends in its indicators in the states of India: the Global Burden of Disease Study 1990–2017. Lancet Child Adolesc Heal. 2019;3(12):855–70. doi: 10.1016/S2352-4642(19)30273-1 31542357 PMC6839043

[22] National Health Mission, Ministry of Health and Family Welfare G of I. Anemia Mukt Bharat. Available from: https://anemiamuktbharat.info/view-your-data-monthly

[23] Government of India. The National Food Security Act, 2013. 2013.

[24] Nguyen PH, Kachwaha S, Pant A, Tran LM, Ghosh S, Sharma PK, et al. Impact of COVID-19 on household food insecurity and interlinkages with child feeding practices and coping strategies in Uttar Pradesh, India: a longitudinal community-based study. BMJ Open. 2021;11(4):e048738. doi: 10.1136/bmjopen-2021-048738 33883156 PMC8061560

[25] World Health Organization. Pulse survey on continuity of essential health services during the COVID-19 pandemic: interim report, 27 August 2020. World Health Organization; 2020. Available from: https://www.who.int/publications/i/item/WHO-2019-nCoV-EHS_continuity-survey-2020.1

[26] Akseer N, Kandru G, Keats EC, Bhutta ZA. COVID-19 pandemic and mitigation strategies: implications for maternal and child health and nutrition. Am J Clin Nutr. 2020 Aug 1;112(2):251–6. doi: 10.1093/ajcn/nqaa171 32559276 PMC7337702

[27] Braun R, Catalani C, Wimbush J, Israelski D. Community health workers and mobile technology: a systematic review of the literature. PLoS One. 2013;8(6):e65772. doi: 10.1371/journal.pone.0065772 23776544 PMC3680423

[28] Ward VC, Raheel H, Weng Y, Mehta KM, Dutt P, Mitra R, et al. Impact of mHealth interventions for reproductive, maternal, newborn and child health and nutrition at scale: BBC Media Action and the Ananya program in Bihar, India. J Glob Health. 2020 Dec;10(2):021005. doi: 10.7189/jogh.10.021005 33425329 PMC7758913

[29] Patil S, Nimmagadda S, Gopalakrishnan L, Bajaj S, Diamond-Smith N, Paul A, et al. mHealth at Scale: Quasi-Experimental Evidence from an Integrated Nutrition Program in India. BMJ Glob Heal, 2022 (in press) 10.1136/bmjgh-2021-007298 PMC9296874 35835476

[30] Galle A, Semaan A, Huysmans E, Audet C, Asefa A, Delvaux T, et al. A double-edged sword-telemedicine for maternal care during COVID-19: findings from a global mixed-methods study of healthcare providers. BMJ Glob Heal. 2021;6(2). doi: 10.1136/bmjgh-2020-004575 33632772 PMC7908054

[31] UNICEF, Indian Institute of Technology Bombay, International Food Policy Research Institute, World Food Programme, World Bank. POSHAN COVID-19 RESOURCES. 2020. Available from: https://poshancovid19.in/

[32] Kohli N, Nguyen PH, Avula R, Menon P. The role of the state government, civil society and programmes across sectors in stunting reduction in Chhattisgarh, India, 2006–2016. BMJ Glob Heal. 2020 Jul 6;5(7):e002274. 10.1136/bmjgh-2019-002274 PMC7342433 32636312

[33] Kohli N, Avula R, van den Bold M, Becker E, Nisbett N, Haddad L, et al. What will it take to accelerate improvements in nutrition outcomes in Odisha? Learning from the past. Glob Food Sec. 2017 Mar;12(October 2016):38–48.

[34] Perry HB, Zulliger R, Rogers MM. Community Health Workers in Low-, Middle-, and High-Income Countries: An Overview of Their History, Recent Evolution, and Current Effectiveness. Annu Rev Public Health. 2014 Mar 18;35(1):399–421. 24387091 10.1146/annurev-publhealth-032013-182354

[35] John A, Nisbett N, Barnett I, Avula R, Menon P. Factors influencing the performance of community health workers: A qualitative study of Anganwadi Workers from Bihar, India. PLoS One. 2020;15(11 November):1–17. doi: 10.1371/journal.pone.0242460 33237939 PMC7688170

[36] Bhaumik S, Moola S, Tyagi J, Nambiar D, Kakoti M. Community health workers for pandemic response: a rapid evidence synthesis. BMJ Glob Heal. 2020 Jun 10;5(6):e002769. doi: 10.1136/bmjgh-2020-002769 32522738 PMC7292038

[37] IDinsight. Four challenges (and solutions) to conducting phone surveys in a refugee settlement. Available from: https://medium.com/idinsight-blog/four-challenges-and-solutions-to-conducting-phone-surveys-in-a-refugee-settlement-6b0ed28d1500

[38] Nguyen P, Kachwaha S, Pant A, Tran LM, Walia M, Ghosh S, et al. Impacts of COVID-19 on Provision and Utilization of Health and Nutrition Services in Uttar Pradesh, India: Insights From Phone Surveys and Administrative Data. Curr Dev Nutr. 2021 Jun 7;5(Supplement_2):672–672. 10.1093/jn/nxab135 PMC8195077 34236434

[39] Our world in data. Overview of the Coronavirus Pandemic (COVID-19). Available from: https://ourworldindata.org/covid-overview

