# Supplementary material for: Disruptions, restorations and adaptations to health and nutrition service delivery in multiple states across India over the course of the COVID-19 pandemic in 2020: An observational study
Source: PLoS One. 2022 Jul 27;17(7):e0269674. doi: 10.1371/journal.pone.0269674 (PMC9328539; doi:10.1371/journal.pone.0269674)
Supplement: S1 Table — Anganwadi worker, Accredited Social Health Activist, and Auxiliary Nurse Midwife are three cadres of frontline workers who are part of India’s national programs that deliver maternal and child nutrition services. “—” No data available. Notes: The exceptionally high response rates in Bihar were because CARE-India has ongoing learning assessments in the state and therefore have established field teams with contacts with FLWs for the past five years. In Chhattisgarh and Tamil Nadu, FLW phone numbers were obtained from the government and most numbers were valid. In Telangana, NIN had prior relationship with ASHAs who were interviewed first, and the trust building helped the team in interviewing AWWs whose phone numbers were obtained from ASHAs. In Madhya Pradesh and Uttar Pradesh, FLWs from studies conducted in 2018 and 2019, respectively, were interviewed and hence there were more unreachable phones, wrong numbers, and refusal rates. The analytic sample includes 4,293 FLWs (Fig 1). As AWWs were the common cadre interviewed in all the states, we present results of AWW surveys; the findings were similar to that of the other cadres (Web appendix). (DOCX) [file pone.0269674.s001.docx]

**S1 Table.** **Characteristics of frontline workers delivering health and nutrition services**

|  | **Bihar** | **Chhattisgarh** | **Madhya Pradesh** | **Odisha** | **Telangana** | **Tamil Nadu** | **Uttar Pradesh** |
| --- | --- | --- | --- | --- | --- | --- | --- |
| Data collection month | **August** | **August** | **September** | **October** | **September** | **September** | **August** |
| ***Anganwadi* Workers** |  |  |  |  |  |  |  |
| Intended sample^1^, n | 1070 | 816 | 711 | 415 | 106 | 500 | 178 |
| Achieved sample, n | 1068 | 642 | 330 | 379 | 99 | 487 | 111 |
| Response rate, % | 99.8 | 78.7 | 46·4 | 91·3 | 93·4 | 97.4 | 62·4 |
| Age, y (mean) | 40·7 | 39·6 | 40·8 | 40·6 | 42·0 | 42 | 45·2 |
| Work duration, y (mean) | 15·1 | 14·8 | 16·8 | 15·1 | 17·0 | 13·0 | 17·3 |
| Have government smart phone, % | 90·1 | 37·8 | 90·8 | 0·0 | 24·7 | 96·5 | 13·6 |
| Have own smart phone, % | 96·9 | 74·7 | 77·0 | 0·8 | 89·9 | 95·1 | 19·8 |
| **Accredited Social Health Activist** |  |  |  |  |  |  |  |
| Intended sample, n | 1069 | -- | -- | 284 | 184 | -- | 174 |
| Achieved sample, n | 1068 | -- | -- | 232 | 102 | -- | 120 |
| Response rate, % | 99.9 | -- | -- | 81·7 | 55·4 | -- | 69·0 |
| Age, y (mean) | 38·5 | -- | -- | 42·6 | 39·0 | -- | 39·6 |
| Work duration, y (mean) | 11·2 | -- | -- | 12·6 | 12·0 | -- | 11·6 |
| Have government smart phone, % | 0·4 | -- | -- | 0·1 | 12·5 | -- | 40·0 |
| Have own smart phone, % | 27·9 | -- | -- | 0·3 | 62·7 | -- | 12·8 |
| **Auxiliary nurse midwife** |  |  |  |  |  |  |  |
| Intended sample, n | 534 | -- | -- | -- | 122 | -- | 127 |
| Achieved sample, n | 534 | -- | -- | -- | 102 | -- | 85 |
| Response rate, % | 100·0 | -- | -- | -- | 83·6 | -- | 66.9 |
| Age, y (mean) | 45·0 | -- | -- | -- | 40·0 | -- | 41·6 |
| Work duration, y (mean) | 15·8 | -- | -- | -- | 14·0 | -- | 14·3 |
| Have government smart phone, % | 31·8 | -- | -- | -- | 14·9 | -- | 59·7 |
| Have own smart phone, % | 72·5 | -- | -- | -- | 94·1 | -- | 67·1 |

^1^Intended sample refers to original sample plan. Anganwadi worker, Accredited Social Health Activist, and Auxiliary nurse midwife are three cadres of frontline workers who are part of India’s national programs that deliver maternal and child nutrition services. “--” No data available

Notes: The exceptionally high response rates in Bihar were because CARE-India has ongoing learning assessments in the state and therefore have established field teams with contacts with FLWs for the past five years. In Chhattisgarh and Tamil Nadu, FLW phone numbers were obtained from the government and most numbers were valid. In Telangana, NIN had prior relationship with ASHAs who were interviewed first, and the trust building helped the team in interviewing AWWs whose phone numbers were obtained from ASHAs. In Madhya Pradesh and Uttar Pradesh, FLWs from studies conducted in 2018 and 2019, respectively, were interviewed and hence there were more unreachable phones, wrong numbers, and refusal rates. The analytic sample includes 4,293 FLWs (Figure 1). As AWWs were the common cadre interviewed in all the states, we present results of AWW surveys; the findings were similar to that of the other cadres (Web appendix).
