# Supplementary material for: Disruptions, restorations and adaptations to health and nutrition service delivery in multiple states across India over the course of the COVID-19 pandemic in 2020: An observational study
Source: PLoS One. 2022 Jul 27;17(7):e0269674. doi: 10.1371/journal.pone.0269674 (PMC9328539; doi:10.1371/journal.pone.0269674)
Supplement: S2 Table — (DOCX) [file pone.0269674.s002.docx]

**S2 Table. Knowledge of frontline workers**

|  | **Bihar** | **Chhattisgarh** | **Madhya Pradesh** | **Odisha** | **Telangana** | **Tamil Nadu** | **Uttar Pradesh** |
| --- | --- | --- | --- | --- | --- | --- | --- |
|  | **N = 1070** | **N = 597** | **N = 330** | **N = 378** | **N = 99** | **N = 487** | **N = 111** |
| Wear a mask while going outside the house | 65·1 | 83·9 | 83·6 | 65·9 | 70·7 | 77·8 | 73·0 |
| Wash hands frequently | 57·9 | 31·7 | 27·9 | 85·5 | 50·5 | 54·8 | 43·2 |
| Wash hands frequently with soap | 81·8 | 53·8 | 67·6 | 90·5 | 72·7 | 74·7 | 68·5 |
| Clean hands with sanitizer | 71·1 | 63·5 | 80·6 | 80·2 | 96·0 | 50·3 | 77·5 |
| Cover nose and mouth with a handkerchief/ tissue/ elbow while coughing or sneezing | 22·3 | 10·7 | 9·1 | 27·3 | 46·5 | 27·7 | 15·3 |
| Avoid crowded places | 38·1 | 31·0 | 34·9 | 56·4 | 49·5 | 45·4 | 41·4 |
| Staying at home as much as possible | 32·2 | 29·0 | 27·6 | 31·8 | 44·4 | 40·2 | 21·6 |
| Maintain physical distance from other people – be at least 1m away | 74·7 | 79·2 | 89·4 | 70·9 | 67·7 | 74·1 | 78·4 |
| Avoid touching face – eyes, nose, mouth | 19·7 | 4·4 | 6·1 | 38·9 | 39·4 | 16·8 | 11·7 |
| Don’t spit in public | 3·9 | 0·5 | 3·0 | 16·7 | 19·2 | 8·4 | 0·9 |
| Wear a mask if sick | 16·8 | 1·5 | 11·2 | 31·0 | 33·3 | 17·0 | 22·5 |
| Avoid coming in physical contact with infected individuals | 15·5 | 3·0 | 16·1 | 33·1 | 21·2 | 8·2 | 11·7 |
| Avoid touching common surfaces, items, plates or utensils | 5·1 | 1·7 | 9·4 | 9·3 | 27·3 | 10·9 | 5·4 |
| Keep cleaning common surfaces | 11·7 | 7·4 | 13·6 | 10·1 | 18·2 | 15·2 | 3·6 |
