# Supplementary material for: Disruptions, restorations and adaptations to health and nutrition service delivery in multiple states across India over the course of the COVID-19 pandemic in 2020: An observational study
Source: PLoS One. 2022 Jul 27;17(7):e0269674. doi: 10.1371/journal.pone.0269674 (PMC9328539; doi:10.1371/journal.pone.0269674)
Supplement: S3 Table — (DOCX) [file pone.0269674.s003.docx]

**S3 Table. *Anganwadi* workers affected by COVID-19 (April 2020)**

|  | **Bihar** | **Madhya Pradesh** | **Odisha** | **Telangana** | **Tamil Nadu** | **Uttar Pradesh** |
| --- | --- | --- | --- | --- | --- | --- |
|  | **N = 1070** | **N = 330** | **N = 378** | **N = 99** | **N = 487** | **N = 111** |
|  | **%** | | | | | |
| Unemployment/loss of income | 60·0 | 37·0 | 42·0 | 37·4 | 42·5 | 64·9 |
| Problems with food (high prices, low availability, low access) | 33·0 | 52·4 | 44·0 | 42·4 | 15·6 | 36·0 |
| Shops being closed | 28·0 | 47·3 | 44·0 | 33·3 | 51·3 | 43·2 |
| Long distance travel restrictions | 24·0 | 23·0 | 33·0 | 30·3 | 32·2 | 21·6 |
| Not visiting family/friends (social distancing) | 19·0 | 12·4 | 8·0 | 33·3 | 19·1 | 12·6 |
| Staying indoors (quarantine/self-quarantine) | 10·0 | 0·3 | 7·0 | 20·2 | 34·9 | 3·6 |
| Household members have gotten sick | 19·0 | 6·4 | 9·0 | 13·1 | 4·7 | 1·8 |
| Fear of household members getting sick | 19·0 | 22·1 | 10·0 | 39·4 | 13·8 | 21·6 |
| Less able to access health services | 8·0 | 5·8 | 7·0 | 16·2 | 2·1 | 6·3 |
| Burden of additional household duties | 11·0 | 4·2 | 8·0 | 14·1 | 9·4 | 8·1 |
| Burden of additional work | 16·0 | 6·1 | 11·0 | 37·4 | 6·2 | 5·4 |
| Travel long distances for work because of lack of transport | 19·0 | 6·7 | 5·0 | 29·3 | 24·4 | 14·4 |
| HH members who were visiting other places got stuck/couldn’t travel back home | 11·0 | 2·7 | 3·0 | 4·0 | 5·1 | 6·3 |

Values are percentage
