# Supplementary figures and images for: Disruptions, restorations and adaptations to health and nutrition service delivery in multiple states across India over the course of the COVID-19 pandemic in 2020: An observational study
Source: PLoS One. 2022 Jul 27;17(7):e0269674. doi: 10.1371/journal.pone.0269674 (PMC9328539; doi:10.1371/journal.pone.0269674)

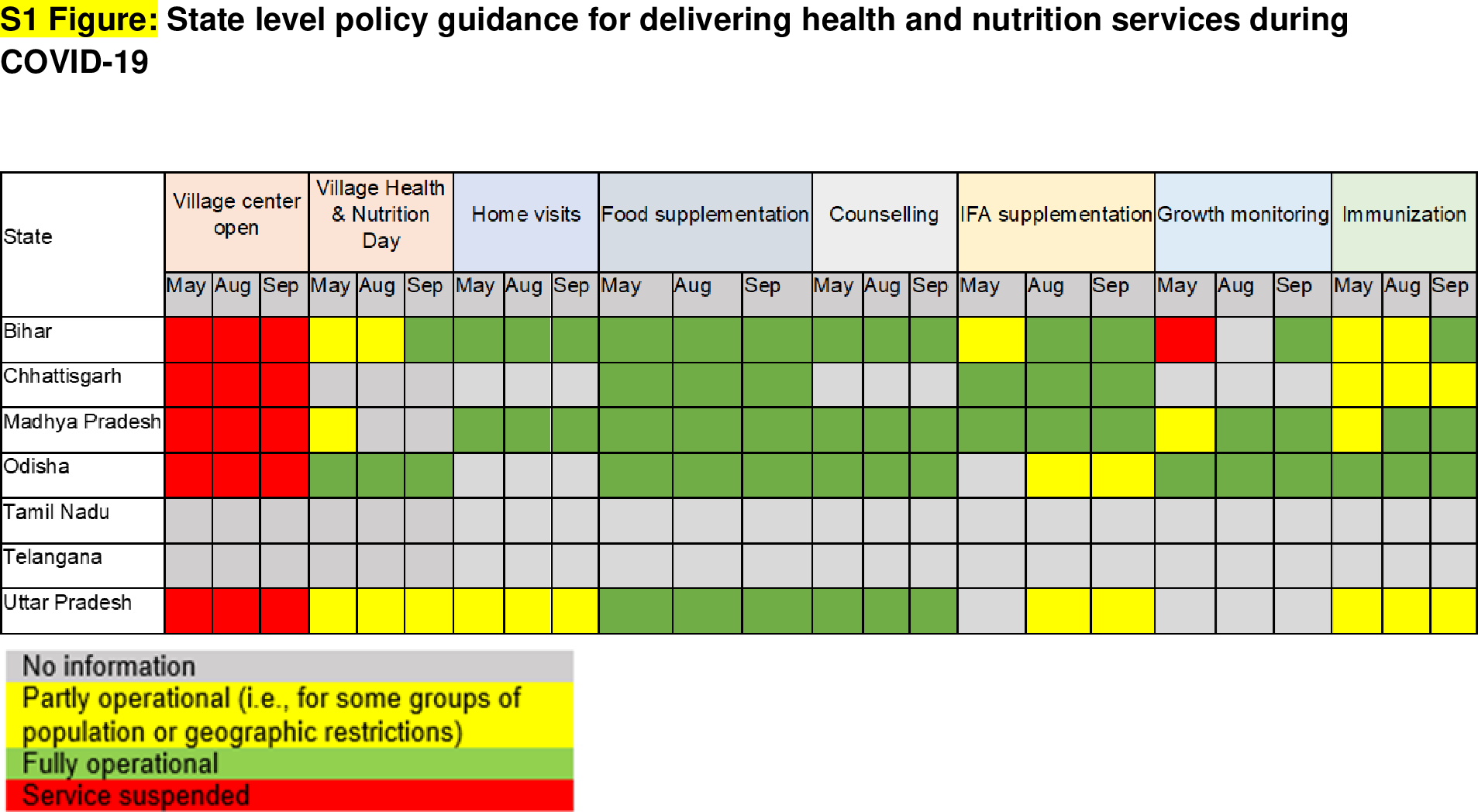

Supplement: S1 Fig — (TIF) [file pone.0269674.s004.tif]

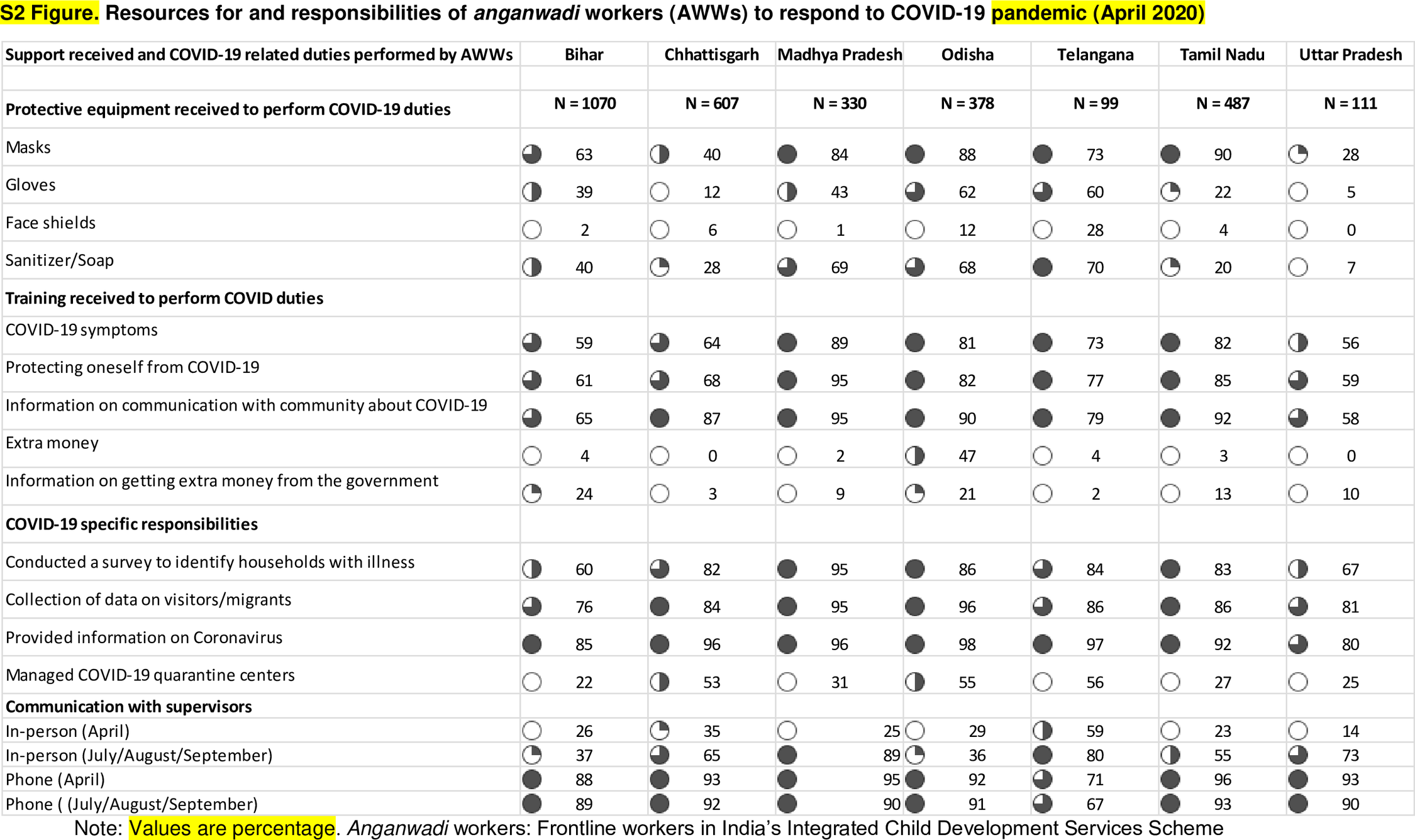

Supplement: S2 Fig — (TIF) [file pone.0269674.s005.tif]
