# Supplementary material for: Disruptions, restorations and adaptations to health and nutrition service delivery in multiple states across India over the course of the COVID-19 pandemic in 2020: An observational study
Source: PLoS One. 2022 Jul 27;17(7):e0269674. doi: 10.1371/journal.pone.0269674 (PMC9328539; doi:10.1371/journal.pone.0269674)
Supplement: S1 File — (DOCX) [file pone.0269674.s006.docx]

**S1 Questionnaire**

**Phone survey with frontline workers (FLWs) in the context of adaptations to health and nutrition service delivery in COVID-19, India**

Table of Contents

[**Module A: Administrative [9 Questions]** 2](#_Toc49160116)

[**Module B: Adaptation to service delivery (25 questions)** 4](#_Toc49160117)

[**Module C: Status of other village-level services [3 question]** 15](#_Toc49160118)

[**Module D: Communication and supervision [5 questions]** 16](#_Toc49160119)

[**Module E: COVID responsibilities [5 questions]** 18](#_Toc49160120)

[**Module F: COVID Knowledge and its influence [4 questions]** 20](#_Toc49160121)

[**Module G: Permission for repeat call [4 Questions]** 23](#_Toc49160122)

# **Module A: Administrative [9 Questions]**

| **Administrative** | **Response** |
| --- | --- |
| 1. Which frontline worker is this?   यह कौन सी कार्यकर्ता हैं? | 1. Anganwadi Worker (AWW) आंगनवाड़ी कार्यकर्ता 2. ASHA आशा   3. ANM |
| 1**.** State Name  राज्य का नाम | Pre-fill |
| 2. District Name  जिला का नाम | Pre-fill |
| 3. Block Name  ब्लॉक का नाम | Pre-fill |
| 4. Village Name  गाँव का नाम | Pre-fill |
| 5. What is your name?  आपका नाम क्या है? |  |
| 6. What is your age?  आपकी उम्र क्या है? | [____] YEARS [Value between 18-60]  [______] साल |
| 7. How long have you been working as **AWW/ASHA/ANM**? **Fill from A0**  आप आंगनवाड़ी कार्यकर्ता/ आशा/ ANM के तौर पर कितने सालों से कम कर रही है? | [______] COMPLETED YEARS [Value <42]  [______] साल |
| ***ASK IF Q0 ==1 \| 2***  8. What is the total number of households in your catchment area?  आपके कार्यक्षेत्र में कुल कितने घर है? | No. of households [Number] __________________  कुल घर [संख्या]____________ |
| 9. Please tell me whether you:  कृपया मुझे बताइए क्या आपके पास: | |
| 1. Do you have your own smart phone (a phone which has internet access)?   अपना स्मार्ट फोन है – (फ़ोन जिसमें इन्टरनेट का इस्तेमाल होता है) | 0. No नहीं  1. Yes हाँ |
| ***ASK IF Q9a ==0***   1. Do you have access to any mobile phone? (your own or family member)?   क्या आपके पास किसी फ़ोन को इस्तेमाल करने की सुविधा है (अपना हो या परिवार के किसी सदस्य का हो) | 0. No नहीं  1. Yes हाँ |
| ***ASK IF Q9a ==1***   1. Did you get this phone from government?   क्या आपको यह फोन सरकार से मिला था? | 0. No नहीं  1. Yes हाँ |
| ***ASK IF Q9a==1 & Qc ==1***   1. Do you use ICDS-CAS on your phone?  क्या आप अपने फ़ोन में ICDS-CAS का इस्तेमाल करती है? | 0. No नहीं  1. Yes हाँ |

# **Module B: Adaptation to service delivery (25 questions)**

**मोड्यूल B: सेवा वितरण का रूपांतर**

*Note: (i) Rows shaded in yellow are new questions; (ii) Rows shaded in green capture adaptions by FLWs*

***For all questions DO NOT READ OPTIONS unless being told to do***

***सभी प्रश्नों के लिए विकल्पों को पढना नहीं है, जब तक की कहा न जाये |***

| ***Interviewer: I will now ask you about services provided during last one month in July and during lockdown in April 2020***  ***इंटरव्यूअर: अब मै आपसे अप्रैल 2020 में कोरोना वायरस लॉक डाउन के दौरान दी गयी सेवाओं के बारे में पूछूंगी\|*** | | | |
| --- | --- | --- | --- |
| **FOR AWWs ONLY (ASK IF MODULE A Q0==1)**   1. Did you open the AWC every day?   क्या आपने आंगनवाडी केंद्र हर रोज़ खोला? | A. In the last month  पिछले एक महीने में क्या आपने आंगनवाडी केंद्र हर रोज़ खोला?  0. Not at all=0  बिलकुल भी नहीं  1. Some days as needed=1  कुछ दिन ज़रुरत के अनुसार  2. Yes, Every day=2 रोजाना | B. During the corona virus lockdown in April  अप्रैल में कोरोनावायरस लॉक डाउन के दौरान क्या आपने आंगनवाडी केंद्र हर रोज़ खोला?  0. Not at all=0  बिलकुल भी नहीं  1. Some days as needed=1  कुछ दिन ज़रुरत के अनुसार  2. Yes, Every day=2 रोजाना | |
| **Village Health sanitation and Nutrition Day (VHSND)** | | | |
| 1. Were you able to help in conducting Village Health and Nutrition Day?   आप VHND (ग्राम स्वास्थ्य एवं पोषण दिवस) का आयोजन कर पायी थी? या आयोजन में सहायता दे पाई ? | A. In the last month  पिछले एक महीने में आप VHND (ग्राम स्वास्थ्य एवं पोषण दिवस) का आयोजन कर पायी थी? या आयोजन में सहायता दे पाई ?  0. No नहीं  1. Yes हाँ | B. During the corona virus lockdown in April  अप्रैल में कोरोनावायरस लॉक डाउन के दौरान आप VHND (ग्राम स्वास्थ्य एवंपोषण दिवस) का आयोजन कर पायी थी? या आयोजन में सहायता दे पाई ?  0. No नहीं  1. Yes हाँ | |
| **ASK IF Q2A==1 \| Q2B==1**   1. How did you manage to organize VHND?   ***Code multiple responses***  VHND का आयोजन कैसे कर पाईं थी?  ***एक से अधिक विकल्पों को कोड करे***  **LOGIC: IF Q3==0, then cannot choose multiple options**  **यदि Q3==0 है तो एक से अधिक उत्तर नहि हो सक्ता** | 1. Conducted VHND as usual  VHND पहले की ही तरह आयोजित किया  2. VHND was conducted for some beneficiaries only  VHND केवल कुछ लाभार्थियों के लिए आयोजित किया गया  3. VHND was organized for different groups of beneficiaries at different times of the day  VHND को दिन में अलग अलग समय पर अलग अलग लाभार्थियों के लिए आयोजित किया  4. VHND session was conducted over multiple days to cover the beneficiaries  सभी लाभार्थियों को सेवा देने के लिए VHND सत्र कई दिनों तक चला  5. All beneficiaries were given masks  सभी लाभार्थियों को मास्क दिया गया  6. Beneficiaries were asked to maintain distance  लाभार्थियों को दूरी बनाये रखने के लिए कहा  7. Marked areas for seating  सबके लिए बैठने की जगह चिन्हित की गयी  8. AWW/ASHA/ANM wore masks  AWW/ASHA/ANM ने मास्क पहना था  9. Kept sanitizer/soap and water ready  सैनीटाईजर या साबुन और पानी तैयार रखे थे  10. Disinfected the VHND premises  VHND की जगह को कीटाणु मुक्त किया / साफ़ सफाई की  95. Other (specify)=95  अन्य (बताएं ) ________________ | | |
| **Family planning services** | | | |
| **FOR ANM/ASHA ONLY (If Q A0==2 \| QA0==3)**  3a. Did you distribute FP products (condoms/oral contraceptive pills) to eligible couple?  **परिबार नियोजन सम्बंधित प्रोडक्ट (कंडोम / गर्भनिरोधक गोली) को आप ने लाभार्थियों को बितरित किया था ?**  **Skip to 3d if 3aA==0 & 3aB==0** | A. In the last month  पिछले एक महीने में क्या आप **परिबार नियोजन सम्बंधित प्रोडक्ट** वितरित कर पायीं थीं?  0. No नहीं  1. Yes हाँ | | B. During the corona virus lockdown in April  अप्रैल में कोरोना वायरस लॉक डाउन के दौरान क्या आप **परिबार नियोजन सम्बंधित प्रोडक्ट** वितरित कर पायीं थीं?  0. No नहीं  1. Yes हाँ |
| **Take-home ration (THR) and hot-cooked meals**  **घर ले जाने वाला राशन ( THR) और गरम पका भोजन** | | | |
| **FOR AWWs ONLY (ASK IF MODULE A Q0==1)**   1. Were you able to provide THR?   क्या आप THR वितरित कर पायीं थीं? | A. In the last month  पिछले एक महीने में क्या आप THR वितरित कर पायीं थीं?  0. No नहीं  1. Yes हाँ  2. Yes, through DBT | | B. During the corona virus lockdown in April  अप्रैल में कोरोना वायरस लॉक डाउन के दौरान क्या आप THR वितरित कर पायीं थीं?  0. No नहीं  1. Yes हाँ |
| **ASK IF Q4B==1**   1. During corona virus lockdown, how did you manage to provide THR?   ***Code multiple responses***  *कोरोना वायरस लॉक डाउन के दौरान* लाभार्थियों को THR कैसे *दे पाए थे?*  ***एक से अधिक विकल्पों को कोड करे*** | 1. As usual at the AWC  पहले की तरह आंगनवाड़ी केंद्र में निश्चित दिनों पर  2. I/AWH delivered THR to beneficiary homes  मै या सहायिका लाभर्थियों के घरों में THR दिया  95. Other (specify)  अन्य (बताएं ) | | |
| **ASK IF Q4B==0**   1. During coronavirus lockdown in April, what did you provide instead of THR?   अप्रैल में कोरोना वायरस लॉक डाउन के दौरान आपने THR की जगह पर क्या दिया? | 1. Nothing कुछ नहीं  2. Provided cash instead of THR  THR की जगह पैसे दिए गए थे  3. Provided dry ration (e.g., rice, dal, wheat etc) instead of THR  THR की जगह सूखा राशन (चावल, दाल, गेहूं) दिया गया था  95. Other (specify) अन्य (बताएं ) __________ | | |
| **FOR AWWs ONLY (ASK IF MODULE A Q0==1)**   1. Were you able to provide hot-cooked meals to children?   क्या आप बच्चों को गरम पका हुआ भोजन/खाना प्रदान कर पाईं थीं? | A. In the last month  पिछले एक महीने में क्या आप बच्चों को गरम पका हुआ भोजन/खाना प्रदान कर पाईं थीं?  0. No नहीं  1. Yes हाँ | | B. During the corona virus lockdown in April  अप्रैल में कोरोना वायरस लॉक डाउन के दौरान क्या आप बच्चों कोगरम पका हुआ भोजन/खाना प्रदान कर पाईं थीं?  0. No नहीं  1. Yes हाँ |
| **ASK IF Q7B==0**   1. During coronavirus lockdown in April, what did you provide instead of hot-cooked meals? (e.g., THR or dry rations)   अप्रैल में कोरोना वायरस लॉक डाउन के दौरान आपने गरम पके हुए भोजन/खाने के बजाये क्या दिया? (जैसे THR या सूखा राशन (चावल, दाल, गेहूं) | 1. Nothing कुछ नहीं  2. Provided THR in lieu of HCM  गरम पके हुए भोजन/खाने के बजाये THR दिया गया था  3. Dry ration (e.g., rice, dal, wheat etc) instead of HCM  सूखा राशन (चावल, दाल, गेहूं) दिया गया था  4. Provided money in lieu of HCM गरम पके हुए भोजन/खाने की जगह पैसे दिए गए  5. Other locally prepared nutritious supplements  अन्य ग्राम के स्तर पे बनाये गए पोषाहार  95. Other (specify) अन्य (बताएं ) | | |
| **Growth monitoring विकास की निगरानी** | | | |
| 1. Were you able to measure weight or height of children?   क्या आप बच्चों के वजन या कद का नाप कर पाईं थीं? | A. In the last month  पिछले एक महीने में क्या आप बच्चोंके वजन या कद कानाप कर पाईं थीं या उसमे मदद कर पायीं थीं?  0. No नहीं  1. Yes हाँ | | B. During the corona virus lockdown in April  अप्रैल में कोरोना वायरस लॉक डाउन के दौरान क्या आप बच्चोंके वजन या कद कानाप कर पाईं थीं या उसमे मदद कर पायीं थीं?  0. No नहीं  1. Yes हाँ |
| 1. Were you able to refer malnourished children to ANMs/ASHAs/nutrition rehabilitation centers? क्या आप अतिकुपोषित बच्चों को आशा, ANM, या NRC को रेफर कर पायीं थीं? | A. In the last month  पिछले एक महीने में क्या आप अतिकुपोषित बच्चों को आशा, ANM, या NRC को रेफर कर पायीं थीं?  0. No नहीं  1. Yes हाँ  2. There were no identified malnourished children in the catchment area  कार्यखेत्र मैं कोई भी कुपोषित बच्चा नही हैं | | B. During the corona virus  lockdown  कोरोना वायरस लॉक डाउन के दौरान क्या आप अतिकुपोषित बच्चों को आशा, ANM, या NRC को रेफर कर पायीं थीं?  0. No नहीं  1. Yes हाँ  2. There were no identified malnourished children in the catchment area  कार्यखेत्र मैं कोई भी कुपोषित बच्चा नही हैं |
| **Immunization टीकाकरण** | | | |
| 1. Were you able to provide or help in providing immunization services for pregnant women and children?   क्या आप गर्भवती महिलाओं और बच्चों का टीकाकरण कर पाईं थीं या उसमे मदद कर पायीं थीं? | A. In the last month  पिछले एक महीने में क्या आप गर्भवती महिलाओं और बच्चों का टीकाकरण कर पाईं थीं या उसमे मदद कर पायीं थीं?  0. No नहीं  1. Yes, for all beneficiaries हाँ, सभी लाभार्थियों को  2. Yes, for pregnant women only हाँ, केवल गर्भवती महिलाओं को  3. Yes, for children only हाँ, केवल बच्चों को | | B. During the corona virus lockdown in April  अप्रैल में कोरोना वायरस लॉक डाउन के दौरान क्या आप गर्भवती महिलाओं और बच्चों का टीकाकरण कर पाईं थीं या उसमे मदद कर पायीं थीं?  0. No नहीं  1. Yes, for all beneficiaries  हाँ, सभी लाभार्थियों को  2. Yes, for pregnant women only  हाँ, केवल गर्भवती महिलाओं को  3. Yes, for children only  हाँ, केवल बच्चों को |
| 1. How were you able to help beneficiaries receive immunization **services** during the lockdown?   ***Code multiple responses***  लॉक डाउन के दौरान, आप लाभार्थियों को टीकाकरण प्राप्त करने में किस तरह से मदद कर पायी थी?  ***एक से अधिक विकल्पों को कोड करे***  **LOGIC: If Q12==0, then cannot choose multiple options**  **यदि Q12==0 है तो एक से अधिक उत्तर नहि हो सक्ता** | 0. Could not help  मदद नहीं कर पाई  1. No one asked for help  किसी ने मदद के लिए पूंछा नहीं  2. Reminded through WhatsApp message/phone call  व्हाट्सएप के द्वारा / फ़ोन करके याद दिलाया गया था  याद दिलाया गया  3. Visited beneficiaries’ home to call for immunization  लाभार्थी के घर जाके टीकाकरण के लिए बुलाई  4. Made an appointment for immunization  टीकाकरण के लिए अपॉइंटमेंट या समय लिया  5. Arranged for transport to visit the immunization venue  टीकाकरण स्थल पर जाने के लिए परिवहन की व्यवस्था की  6. Coordinated with AWW/ASHA/ANM to arrange for the visit at the immunization venue  आंगनवाड़ी कार्यकर्ता, ASHA या ANM से संपर्क करके टीकाकरण स्थल पर जाने के लिए व्यवस्था की  7. Coordinated with supervisor to arrange for the visit at the immunization venue  सुपरवाइजर से संपर्क करके टीकाकरण स्थल पर जाने के लिए व्यवस्था की  95. Other (specify)________________  अन्य (बताएं ) | | |
| **Antenatal care services (ANC) प्रसव पूर्व देखभाल** | | | |
| 1. Were you able to provide or help in providing antenatal care services to all pregnant women?     क्या आप सभी गर्भवती महिलाओं को प्रसवपूर्व देखभाल की सेवाएं दे पाई थी या उसमे मदद कर पाई थी ? | A. In the last month  पिछले एक महीने में क्या आप सभी गर्भवती महिलाओं को प्रसवपूर्व देखभाल की सेवाएं दे पाई थी या उसमे मदद कर पाई थी ?  0. No one नहीं  1. Yes, only to some pregnant women in the last trimester or for high-risk pregnant women  हाँ, केवल kuch अंतिम तिमाही या गंभीर जोखिम वाली गर्भवती महिलाओं को  2. Yes, to all pregnant women  हाँ, सभी garbhawati महिलाओं को  95. Other (specify) | | B. During the corona virus lockdown in April  अप्रैल में कोरोना वायरस लॉक डाउन के दौरान क्या आप सभी गर्भवती महिलाओं को प्रसवपूर्व देखभाल की सेवाएं दे पाई थी या उसमे मदद कर पाई थी ?  0. No one नहीं  1. Yes, only to some pregnant women in the last trimester or for high-risk pregnant women  हाँ, केवल kuch अंतिम तिमाही या गंभीर जोखिम वाली गर्भवती महिलाओं को  2. Yes, to all pregnant women  हाँ, सभी garbhawati महिलाओं को  95. Other (specify) |
| 1. How were you able to help pregnant women in receiving **antenatal care services** at a health facility or the other fixed site during the lockdown?   ***Code multiple responses***  लॉक डाउन के दौरान, आप गर्भवती महिलाओं को प्रसवपूर्व देखभाल की सेवाएं स्वास्थ केंद्र या अन्य जगह पर प्राप्त करने में किस प्रकार से मदद कर पाईं थी?  ***एक से अधिक विकल्पों को कोड करे***  **LOGIC: If Q14==0, then cannot choose multiple options**  यदि Q14==0 है तो एक से अधिक उत्तर नहि हो सक्ता | 0. Could not help  मदद नहीं कर पाई  1. No one asked for help  किसी ने मदद के लिए पूंछा नहीं  2. Made an appointment for ANC at the health center  प्रसवपूर्व देखभाल के लिए स्वास्थ केंद्र पर अपॉइंटमेंट या समय लिया  3. Arranged for transport to visit the health facility  स्वास्थ केंद्र तक जाने के लिए परिवहन की व्यवस्था कीं  4. Coordinated with ASHA and ANM to arrange for the visit at the health center  ASHA और ANM से संपर्क करके स्वास्थ केंद्र पर जाने के लिए व्यवस्था कीं  5. Coordinated with supervisor to arrange for the visit to the health facility  सुपरवाइजर से संपर्क करके स्वास्थ केंद्र पर जाने के लिए व्यवस्था कीं  6. Reminded through WhatsApp message/phone call  व्हाट्सएप के द्वारा / फ़ोन करके याद दिलाया गया था  7. Visited beneficiaries’ home to call for ANC  लाभार्थी के घर जाके ANC के लिए बुलाई  95. Other specify___  अन्य (बताएं ) | | |
| **IFA supplements IFA पूरक** | | | |
| 1. Were you able to distribute or help in distributing IFA tablets/syrup to:   क्या आप ___को IFA गोली या सिरप बाँट सकीं या बाटने में मदद कर सकीं: | A. In the last month  पिछले एक महीने में क्या आप ___को IFA गोली या सिरप बाँट सकीं या बाटने में मदद कर सकीं:  0. No नहीं  1.Yes, to a few beneficiaries  2. Yes, all beneficiaries  3. No, stock out  -99. No such beneficiary in my area | | B. During the corona virus lockdown in April  अप्रैल में कोरोना वायरस लॉक डाउन के दौरान क्या आप ___को IFA गोली या सिरप बाँट सकीं या बाटने में मदद कर सकीं:  0. No नहीं  1. Yes, to a few beneficiaries  2. Yes, all beneficiaries  3. No, stock out  -99. No such beneficiary in my area |
| 1. Pregnant women   गर्भवती महिलाओं को |  | |  |
| 1. Lactating women   धात्री महिलाओं को |  | |  |
| 1. Children   बच्चों को |  | |  |
| 1. Adolescent girls   किशोरी बालिकाओं को |  | |  |
| **ASK IF Q15Ba\| Q15Bb\| Q15Bc \|Q15Bd==1 or 2**   1. During corona virus lockdown, how did you manage to distribute IFA to beneficiaries?   ***Code multiple responses***  कोरोना वायरस लॉक डाउन के दौरान लाभार्थियों को IFA कैसे बांटे थे?  ***एक से अधिक विकल्पों को कोड करे*** | 2. I/AWH delivered IFA to beneficiary homes  मैंने या सहायिका ने IFA लाभर्थियों के घरों में दिया था  3. Beneficiaries were asked to collect it from AWC  लाभर्थियों को आंगनवाड़ी केंद्र से IFA लेने के लिए कहा गया था  4. Beneficiaries were asked to collect it from health facilities  लाभर्थियों को स्वास्थ केंद्र से लेने के लिए कहा गया था  5. Other FLW/ community volunteer delivered it to beneficiary homes  अन्य कार्यकर्ताओं या सामुदायक स्वयंसेवकों ने लाभर्थियों के घरों में दिया  95. Other (specify)  अन्य (बताएं )_________ | | |
| **Institutional deliveries संस्थागत प्रसव** | | | |
| 1. Please tell us how were you able to help women who delivered during the lockdown?   ***Code multiple responses***  लॉक डाउन के दौरान, महिलाओं के प्रसव के लिए आप किस प्रकार मदद कर पायीं थी?  ***एक से अधिक विकल्पों को कोड करे***  **LOGIC: If Q17==0, then cannot choose multiple options**  यदि Q17==0 है तो एक से अधिक उत्तर नहि हो सक्ता | 0. No deliveries  प्रसव नहीं हुए  1. Did not know about deliveries until after the delivery  जन्म देने के बारे में प्रसव के बाद तक पता नहीं चला  2. I was not able to help  मै मदद नहीं कर पायीं  3. Accompanied to the hospital  साथ में अस्पताल गयी  4. Arranged for transport to the hospital  अस्पताल जाने के लिए गाडी की व्यवस्था की  5. Coordinated with AWW, ASHA or ANM to accompany them to the hospital  आंगनवाड़ी कार्यकर्ता, आशा या ANM को उसके साथ अस्पताल जाने के लिए संपर्क किया  6. Visited home in case of home delivery  घर में प्रसव होने पर घर पर विजिट किया  7. Facilitated child delivery in case of home delivery  घर में प्रसव होने पर, बच्चा पैदा कराया  95. Other (specify) ______ | | |
| **Home visits गृह भ्रमण** | | | |
| 1. Were you able to conduct home visits?   क्या आप गृह भैट कर पायीं थीं? | A. In the last month  पिछले एक महीने में क्या आप गृह भैट कर पायीं थीं?  0. No नहीं  1. Yes हाँ | | B. During the corona virus lockdown in April  अप्रैल में कोरोना वायरस लॉक डाउन के दौरान क्या आप गृह भैट कर पायीं थीं?  0. No नहीं  1. Yes हाँ |
| **ASK IF Q18B==1**  During corona virus lockdown, what did you do during home visits?  ***Code multiple responses***  कोरोना वायरस लॉकडाउन जब चल रहा था, तब आप गृह भैट पे क्यों जाती थी?  ***एक से अधिक विकल्पों को कोड करे*** | To inform about Coronavirusकोरोना वायरस के बारे में जानकारी देने के लिए  To update household listing with migrant population घर में रहने वाले सदस्यों की लिस्ट/सूची में प्रवासी लोगों के नाम जोड़ने के लिए  To check about Coronavirus symptoms कोरोनावायरस के लक्षण की जांच करने के लिए  To distribute THR THR बाँटने के लिए  To distribute IFA IFA बाँटने के लिए  To distribute calcium कैल्शियम बाँटने के लिए  To check well-being of pregnant women गर्भवती महिला की तबीयत पूछने के लिए/ जांच करने के लिए  To counsel about child feeding/childcare बच्चों को खिलाने/ उनके देखभाल के बारे में सलाह देने के लिए  95. Other (specify) अन्य (बताएं) | | |
| **Counselling of beneficiaries लाभार्थियों को परामर्श** | | | |
| 1. Were you able to counsel women on health and nutrition **or** breastfeeding/complementary feeding practices?   क्या आप महिलाओं को स्वास्थ्य और पोषण या स्तनपान / उपरी आहार देने के तरीकों पर सलाह दे पायी थीं? | A. In the last month  पिछले एक महीने में क्या आप महिलाओं को स्वास्थ्य और पोषण या स्तनपान / उपरी आहार देने के तरीकों पर सलाह दे पायी थीं?  0. No नहीं  1. Yes हाँ | | B. During the corona virus lockdown in April  अप्रैल में कोरोनावायरस लॉक डाउन के दौरान क्या आप महिलाओं को स्वास्थ्य और पोषण या स्तनपान / उपरी आहार देने के तरीकों पर सलाह दे पायी थीं?  0. No नहीं  1. Yes हाँ |
| 1. During corona virus lockdown, how did you manage to counsel women on health and nutrition **or** breastfeeding/complementary feeding practices?   ***Code multiple responses***  कोरोना वायरस लॉकडाउन के दौरान, आप महिलाओं को स्वास्थ्य और पोषण या स्तनपान / उपरी आहार देने के तरीकों पर सलाह कैसे या किन माध्यमों से दी पायी थी?  ***एक से अधिक विकल्पों को कोड करे*** | 1. During home visits  गृह भ्रमण के दौरान  2. During ANC/PNC visit  ANC/PNC विजिट के दौरान  3. At a community event/VHND  सामुदायिक कार्यक्रम या VHND के समय  4. Using a phone  फ़ोन कॉल द्वारा  95. Other (specify)____  अन्य (बताएं ) | | |
| **ASK IF Q21==4**  How did you use your phone to counsel women?  आपने फ़ोन का उपयोग कैसे किया? | | | |
| 1. make an Audio call   क्या आपने फ़ोन पर बात करि | 0. No नहीं  1. Yes हाँ | | |
| 1. send a SMS message   क्या आपने फ़ोन पर SMS करि | 0. No नहीं  1. Yes हाँ | | |
| **ASK IF AQ9A==1**   1. send Audio messages on WhatsApp   क्या आपने Whatapp पर ऑडियो मैसेज भेजा | 0. No नहीं  1. Yes हाँ | | |
| **ASK IF AQ9A==1**   1. make a Video call   क्या आपने फ़ोन पर वीडियो पर बात करि | 0. No नहीं  1. Yes हाँ | | |
| **ASK IF AQ9A==1**   1. send Video messages on WhatsApp groups   क्या आपने Whatapp पर वीडियो मैसेज भेजा | 0. No नहीं  1. Yes हाँ | | |
| **ORS and Zinc supplements ORS और जिंक पूरक** | | | |
| 1. Were you able to provide or help in providing ORS/ORS and Zinc to children with diarrhea?   क्या आप डायेरिया से पीड़ित बच्चे को ORS या ORS और ZINC दे पायीं थीं या दिलवाने में मदद कर पायीं थीं? | A. In the last month  पिछले एक महीने में क्या आप डायेरिया से पीड़ित बच्चे को ORS या ORS और ZINC दे पायीं थीं या या दिलवाने में मदद कर पायीं थीं?  0. No नहीं  1. Yes हाँ | | B. During the corona virus lockdown in April  अप्रैल में कोरोना वायरस लॉक डाउन के दौरान क्या आप डायेरिया से पीड़ित बच्चे को ORS या ORS और ZINC दे पायीं थीं या या दिलवाने में मदद कर पायीं थीं?  0. No नहीं  1. Yes हाँ |
| **ASK IF Q22B==1**   1. During the corona virus lockdown in April how did you manage to provide help for children with diarrhoea?   ***Code multiple responses***  अप्रैल में कोरोना वायरस लॉक डाउन के दौरान डायेरिया से पीड़ित बच्चे को आप कैसे मदद कर पायी थी?  ***एक से अधिक विकल्पों को कोड करे*** | 2. I/AWH delivered ORS/ORS and zinc to beneficiary homes  मैंने या सहायिका ने इसे लाभर्थियों के घरों में दिया  3. Beneficiaries collected ORS/ORS and zinc from AWC  लाभार्थियों ने आंगनवाड़ी केंद्र से लिया  4. Beneficiaries collected ORS/ORS and zinc from my house  लाभार्थियों ने मेरे घर से लिया  5. Beneficiaries collected ORS/ORS and zinc from health facilities  लाभार्थियों ने स्वास्थ्य केंद्र से लिया  6. Other FLW or community volunteer delivered ORS/ORS and zinc to beneficiary homes  अन्य कार्यकर्ताओं या सामुदायिक स्वयंसेवकों ने लाभार्थियों के घरों में दिया  7. I told beneficiaries how to prepare ORS at home  लाभार्थियों को घर पे ORS बनाने की तरीका बताई  95. Other specify _______अन्य ( बताएं ) | | |
| 1. What challenges did you face in providing services during lockdown? *(e.g., personal, work-related)*   ***Code multiple responses***  लॉकडाउन के दौरान लोगों को सेवाएं देने में आपको किन-किन दिक्कंतों का का सामना करना पड़ा? (जैसे परिवार से सम्बंदित या काम से जुड़े)  ***एक से अधिक विकल्पों को कोड करे***  **Logic: If Q24==0, then cannot choose multiple options**  **यदि Q24==0 है तो एक से अधिक उत्तर नहि हो सक्ता** | 0. No challenges  कोई परेशानी नहीं हुई  1. There was no transport to reach beneficiaries  लाभार्थियों तक पहुचने के लिए कोई परिवहन नहीं था  2. I had to walk long distances  मुझे लम्बी दूरी तक पैदल चलना पड़ता था  3. I did not receive money to buy THR or dry ration  मुझे THR या सूखा राशन लेने के लिए पैसे नहीं मिले  4. I did not receive THR or dry ration /did not have enough food for all beneficiaries  मुझे THR या सूखा राशन नहीं मिला  5. Beneficiaries did not want me/AWH to come to their house  लाभार्थी नहीं चाहते थे की मै या सहायिका उनके घर आयें  6. Beneficiaries did not want to eat food from AWC during coronavirus  लाभार्थी कोरोना वायरस के दौरान आंगनवाड़ी केंद्र का भोजन नहीं खाना चाहते थे  7. I was scared to go to deliver food at homes because I might contract the virus  मुझे घर-घर जाकर खाना देने में कोरोना वायरस संक्रमण होने का डर था  8. I was sick so could not work  मै बीमार थी इसलिए काम नहीं कर सकी  9. I was busy with coronavirus work  मै कोरोना वायरस के कार्य में व्यस्त थी इसलिए खाना देने नहीं जा सकी 10. Discomfort due to weather/ mask wearing  मौसम/ मास्क लगाने की वजह से दिक्कत होती है   1. I did not have phone or phone broke; so could not communicate with supervisors or beneficiaries   मेरे पास फ़ोन नहीं था/टूट गया था इसीलिए लाभार्थियों या सुपरवाईजर से बात नहीं हो पाई   1. My family members did not want me to work during lockdown मेरे घर के सदस्य नहीं चाहते थे कि मैं लॉकडाउन के दौरान काम करूँ 2. Lot of pressure from my supervisor to do the work सुपरवाईजर की तरफ से काम करने का बहुत दबाव था 3. Did not receive salary /incentives/ honorarium सैलरी/प्रोत्साहन राशि/ मानदेय नहीं मिला 4. Did not have mask, gloves to do my work काम करने के लिए मास्क/ग्लव्स नहीं थे 5. Village/community was angry with me गाँव/समुदाय के लोग मुझसे गुस्सा थे 6. Supply issue/stock out   95. Other (specify)अन्य (बताएं ) | | |
| 1. During this corona virus, what help do you need to provide better services (e.g., supplies, training, anything else)? ***Code multiple responses***   अब यह जो करोना वायरस चल रहा है, इसके दौरान आप को अपने काम को बेहतर तरीके से करने में क्या मदद चाहिए होगा? (जैसे सन्साधन, प्रशिक्षण या और कुछ)  ***एक से अधिक विकल्पों को कोड करे*** | 1. Training on precautions to take  किन बातों का ध्यान देना है उसपर प्रशिक्षण/ट्रेनिंग  2. Masks, gloves, sanitizer मास्क/ग्लव्स/ सैनीटाईजर  3. Travel support (money to cover travel cost) यात्रा करने में सहायता (यात्रा करने में लगने वाले पैसों की भरपाई)  4. Transportation to make home visits गृह भ्रमण करने के लिए परिवहन/ यातायात के साधन  5. Cooperation from the beneficiaries लाभार्थियों द्वारा सहयोग  6. Incentives प्रोत्साहन राशि/ इंसेंटिव  7. Phone recharge to call beneficiaries to set-up home visit appointments लाभार्थी को फ़ोन करके घर पर विजिट प्लान करने के लिए मोबाइल फ़ोन रिचार्ज  8. Ensure there are no supply gaps for THR  सुनिश्चित करें कि THR के लिए सप्लाई/आपूर्ति कम नहीं हैं  9. Ensure there are no supply gaps for IFA  सुनिश्चित करें कि IFA के लिए सप्लाई/आपूर्ति कम नहीं हैं  10. Need support for supervisors  सुपरवाईजर की सहयोग  95. Other (specify) अन्य (बताएं) | | |

# **Module C: Status of other village-level services [3 question]**

**मोड्यूल C : अन्य ग्राम-स्तर सेवाओं की स्थिति**

**ASK ONLY FOR AWWS (ASK IF QA0==1); DO NOT ASK FOR ASHA/ANM**

| 1. **In the last month**, did the (primary/middle) school children receive hot cooked meal in your village?  पिछले एक महीने में क्या (primary/middle) स्कूल के बच्चों को गरम पका हुआ भोजन मिला aapke gaon me? | 0. No नहीं  1. Yes हाँ  99. Don’t know |
| --- | --- |
| **ASK IF QC1==0**  2. Did the children receive dry rations? | 0. No नहीं  1. Yes हाँ  99. Don’t know |
| **ASK IF QC1==0**  3. Did the children receive cash/transfer in bank account? | 0. No नहीं  1. Yes हाँ  99. Don’t know |
| 4. During **the corona virus lockdown in April**, did the school children receive hot cooked meal? | 0. No नहीं  1. Yes हाँ  99. Don’t know |
| **ASK IF QC4==0**  5. Did the children receive dry rations? | 0. No नहीं  1. Yes हाँ  99. Don’t know |
| **ASK IF QC4==0**  6. Did the children receive cash /transfer in bank account? | 0. No नहीं  1. Yes हाँ  99. Don’t know |

# **Module D: Communication and supervision [5 questions]**

**मोड्यूल D: कम्युनिकेशन और सुपरविज़न**

| 1. What were the common ways in which you interacted with other AWWs/ASHAs/ANMs?   ***Code multiple responses***  अपने अन्य AWW /ASHA /ANM से बात करने का सामान्य माध्यम क्या था?  ***एक से ज्यादा विकल्प चुने***  **IF QD1==0, then cannot choose multiple options**  यदि QD1==0 है, तो एक से अधिक उत्तर नहि हो सक्ता | A. In the last month  पिछले एक महीने में अन्य AWW /ASHA /ANM से किन माध्यमों से बात करती थीं?  0. No contact कोई संपर्क नहीं  1. Met in person व्यक्तिगत रूप से मिली  2. Phone फ़ोन पे  95. Other specify (_______) अन्य बताएं | B. During the corona virus lockdown in April  अप्रैल में कोरोना वायरस लॉक डाउन के दौरान कोरोना वायरस लॉक डाउन के दौरान, अन्य AWW /ASHA /ANM लोगों से किन माध्यमों से बात करती थीं?  0. No contact कोई संपर्क नहीं  1. Met in person व्यक्तिगत रूप से मिली  2. Phone फ़ोन पे  95. Other specify (_______) अन्य बताएं |
| --- | --- | --- |
| 1. What were the common ways in which you interacted with your beneficiaries?   ***Code multiple responses***  अपने लाभार्थियों से बात करने का सामान्य माध्यम क्या था?  ***एक से ज्यादा विकल्प चुने***  **IF QD2==0, then cannot choose multiple options**  यदि QD2==0 है तो एक से अधिक उत्तर नहि हो सक्ता | A. In the last month  पिछले एक महीने में अपने लाभार्थियों से बात करने का सामान्य माध्यम क्या था?  0. No contact कोई संपर्क नहीं  1. Met in person व्यक्तिगत रूप से मिली  2. Phone फ़ोन पे  95. Other specify (_______) अन्य बताएं | B. During the corona virus lockdown in April  अप्रैल में कोरोना वायरस लॉक डाउन के दौरान अपने लाभार्थियों से बात करने का सामान्य माध्यम क्या था?  0. No contact कोई संपर्क नहीं  1. Met in person व्यक्तिगत रूप से मिली  2. Phone फ़ोन पे  95. Other specify (_______) अन्य बताएं |
| 1. What were the common ways in which you interacted with your supervisor?   ***Code multiple responses***  आपने अपने सुपरवाइजर से या किन माध्यमों से बात करी थी?  ***एक से ज्यादा विकल्प चुने***  **IF QD3==0, then cannot choose multiple options**  यदि QD3==0 है, तो एक से अधिक उत्तर नहि हो सक्ता | A. In the last month  पिछले एक महीने में आपने अपने सुपरवाइजर से या किन माध्यमों से बात करी थी?  0. No contact कोई संपर्क नहीं  1. Met in person व्यक्तिगत रूप से मिली  2. Phone फ़ोन पे  95. Other specify (_______) अन्य बताएं | B. During the corona virus lockdown in April  अप्रैल में कोरोना वायरस लॉक डाउन के दौरान आपने अपने सुपरवाइजर से या किन माध्यमों से बात करी थी?  0. No contact कोई संपर्क नहीं  1. Met in person व्यक्तिगत रूप से मिली  2. Phone फ़ोन पे  95. Other specify (_______) अन्य बताएं |
| **ASK IF QD3A==1\| QD3B==1**   1. Please tell me what is discussed during these interactions? (e.g., personal problems or work problems)   ***Code multiple responses***  कृपया बताईये कि सुपरवाईजर के साथ इन बातचीत के दौरान किन बातों पे चर्चा होती है ?  **एक से ज्यादा विकल्प चुने** | 1. Update on Coronavirus work   कोरोना वाइरस के कार्यों का अपडेट   1. Update on regular services   नियमित सेवाओं का अपडेट   1. Check on completion of records   चेक किया कि रिकॉर्ड पूरा है   1. Discuss my problems with workload 2. Discuss problems I am facing in the community 3. Discuss problems I am facing at home 4. Discuss about salary and incentives 5. Resumption of services   95. Other अन्य (बताएं) | |

# **Module E: COVID responsibilities [5 questions]**

**मोड्यूल E : कोविड की जिम्मेदारियाँ**

**INTERVIEWER: Readout ALL the options unless except when there is an instruction [DO NOT READ OPTIONS]**

इंटरव्यूअर: सभी विकल्पों को पढ़ें ,जब तक निर्देश न दिया हो की [विकल्पों को न पढ़ें]

| **Question** | **Response** |
| --- | --- |
| 1. Since the corona virus started, please tell us if you _____________?   कृपया मुझे बताइए, कोरोना वायरस जब से शुरू हुआ, , क्या आप ________ | |
| 1. Conducted a survey to identify households with illness   बीमारी से पीड़ित घरों को पहचान ने के लिए के लिए सर्वे करि | 0. No नहीं  1. Yes हाँ |
| 1. Collection of data on visitors/migrants   विजिटरस/ प्रवासीयों का डाटा इक्कठा किया | 0. No नहीं  1. Yes हाँ |
| 1. Provide information on Coronavirus   कोरोना वायरस की जानकारी देना | 0. No नहीं  1. Yes हाँ |
| 1. Manage COVID quarantine centers   कोरोना सेंटर परिचालित किए/ परिचालन में सहयोग किये | 0. No नहीं  1. Yes हाँ |
| 1. To do your work, did you ever (from April onwards) get any of the following from the government?   आप को अपना काम करने के लिए, क्या आपको निम्नलिखित में से कुछ भी सरकार से मिला था? | |
| 1. Masks मास्कस | 0. No नहीं  1. Yes हाँ |
| b. Gloves ग्लव्स | 0. No नहीं  1. Yes हाँ |
| c. Face shields फेस शील्ड | 0. No नहीं  1. Yes हाँ |
| d. Sanitizer सैनिटाइज़र/Soap साबुन | 0. No नहीं  1. Yes हाँ |
| 3. To perform Coronavirus duties, did you receive:  कोरोनावायरस से सम्बंदित ड्यूटी करने के लिए, क्या आपको | |
| 1. Training or guidelines or instruction on the Coronavirus symptoms   कोरोनावायरस के चिह्न की पहचान के लिए प्रशिक्षण या निर्देश मिला था? | 0. No नहीं  1. Yes हाँ |
| 1. Training on how to protect yourself from Coronavirus   कोरोना वायरस से खुद को सुरक्षित रखने के लिए प्रशिक्षण मिला था? | 0. No नहीं  1. Yes हाँ |
| 1. Information on what to communicate with beneficiaries/community about Coronavirus   कोरोना वायरस के बारे में लाभार्थियों को क्या बताना है, इस पर जानकारी मिली थी? | 0. No नहीं  1. Yes हाँ |
| 4. To perform Coronavirus duties, did you:  कोरोनावायरस से सम्बंदित ड्यूटी करने के लिए, क्या आपको | |
| a. receive extra money from the government?  सरकार से कोई अतिरिक्त पैसा मिला? | 0. No नहीं  1. Yes हाँ  -99. DK/No response पता नहीं/कोई जवाब नहीं |
| **ASK IF QE4a==0**  b. receive information that you will get extra money from the government  सरकार से कोई अतिरिक्त पैसों के बारें में कोई जानकारी दी गयी | 0. No नहीं  1. Yes हाँ  -99. DK/No response पता नहीं/कोई जवाब नहीं |
| 5. How do you feel about doing the Coronavirus duties? Do you feel…  आप कोरोना वायरस ड्यूटी करने में कैसा महसूस करती हैं? क्या आपको लगता है कि .... | |
| 1. your workload has increased because of Coronavirus duties   कोरोना वायरस ड्यूटी की वजह से आपका कार्यभार बढ़ गया है | 0. No नहीं  1. Yes हाँ  -99. DK/No response पता नहीं/कोई जवाब नहीं |
| 1. worried/scared that you might get Coronavirus   चिंता/या डर कि आपको कोरोना वायरस हो सकता है? | 0. No नहीं  1. Yes हाँ  -99. DK/No response पता नहीं/कोई जवाब नहीं |

#

# **Module F: COVID Knowledge and its influence [4 questions]**

**मोड्यूल F : COVID की जानकारी और उसका प्रभाव**

**INTERVIEWER: Readout ALL the options unless except when there is an instruction [DO NOT READ OPTIONS]**

इंटरव्यूअर: सभी विकल्पों को पढ़ें ,जब तक निर्देश न दिया हो की विकल्प नहीं पढना[विकल्पों को न पढ़ें]

| **Question** | **Response** |
| --- | --- |
| *I would like to ask you a few questions about what you know about Corona virus. This is so that we can understand what communities think and what maybe some doubts. We are happy to answer any of your questions at the end*  *आप कोरोना वायरस के बारे में क्या जानती है ,मैं अब इस बारे में कुछ प्रश्न पूछना चाहूँगीI यह इसलिए है ताकि हम समझ सकें कि समुदाय क्या सोचता है और कुछ शंकाएं क्या हैI हमें अंत में आपके किसी भी सवाल का जवाब देने में ख़ुशी होगीI* | |
| **1.** What are the methods of protection against the Coronavirus virus?  ***Code multiple responses***  कोविड-19 वायरस से सुरक्षा/बचाव के क्या-क्या तरीकें हैं?  ***एक से ज्यादा विकल्प चुने*** | 1. Wash hands frequently   बार-बार हाथ धोना   1. Wash hands frequently with soap   बार-बार साबुन से हाथ धोना   1. Clean hands with sanitizer   सैनिटाइज़र से हाथ साफ करना   1. Cover nose and mouth with a handkerchief/ tissue/ elbow while coughing or sneezing   खांसते/ छींकते समय नाक और मुँह को रुमाल/ टिश्यू/कोहनी से ढकना   1. Avoid touching face – eyes, nose, mouth   चेहरा- आँख, नाक, मुँह छूने से बचना   1. Maintain physical distance from other people – be at least 1m away   दूसरे व्यक्ति से दूरी बना कर रहना- कम से कम 1 मीटर की दूरी   1. Avoid crowded places   भीड़ वाली जगहों से बचना   1. Don’t spit in public   सार्वजानिक रूप से ना थूकना   1. Wear a mask while going outside the house   घर से बाहर निकलते समय मास्क पहनना   1. Wear a mask if sick   बीमार होने पर मास्क पहनना   1. Avoid coming in physical contact with infected individuals   संक्रमित व्यक्ति के संपर्क में आने से बचना   1. Avoid touching common surfaces, items, plates or utensils   सामूहिक सतहों, वस्तुओं, प्लेट या बर्तनों को छूने से बचना   1. Keep cleaning common surfaces   सामूहिक सतहों को साफ रखना   1. Staying at home as much as possible   जितना संभव हो घर में रहना  95. Other specify (__________) अन्य (बताएं ) |
| 2. During the Coronavirus pandemic, should mothers breastfeed their children?  कोरोना महामारी के दौरान क्या माताओं को स्तनपान करना चाहिए? | 0. No नहीं  1. Yes हाँ  -99. DK/No response पता नहीं/कोई जवाब नहीं |
| **3.** Where did you hear about how to prevent coronavirus?  ***Code multiple responses***    आपने कोरोना वायरस के रोकथाम के तरीकों के बारे में कहाँ-कहाँ सुना था?  ***एक से ज्यादा विकल्प चुने*** | 1. From the WCD/health department staff or notifications   WCD/स्वास्थ्य विभाग के कर्मचारियों या नोटिफिकेशन/सूचनाओं से  2. ASHA आशा/AWWs/ANM  3. TV टीवी  4. Radio रेडियो  5. Print and Poster प्रिंट और पोस्टर  6. Audiovan/ speaker ऑडियो वैन/ स्पीकर  7. Voice message received through a phone call (IVR)  फोन कॉल (आईवीआर) से ध्वनि सन्देश मिला  8. Voice message heard when calling someone (ring tone)  किसी को कॉल करते समय(रिंगटोन) ध्वनि सन्देश सुना  9. Personal SMS/Whatsapp groups  पर्सनल एसएमएस/ व्हाट्सऐप ग्रुप  10. Friends/family/community member  दोस्त/ परिवार/ सामुदायिक सदस्य  11. SMS/WhatsApp group from dept.  95. Other specify (__________)  अन्य ( बताएं )  -99. Don’t know/ Refuse to answer  पता नहीं/ जवाब देने से मना किया |
| 4. During lockdown people have faced difficulties. How was your household affected?  ***Code multiple responses***  लोखड़ौन की वजह से कई लोगों पर तरह तरह के प्रभाव पढ़े थे\| क्या आप के घर परिवार पर भी क्या कोई प्रभाव पढ़ा था  ***एक से ज्यादा विकल्प चुने*** | 1. Unemployment/loss of income   बेरोजगारी/ आय का नुकसान   1. Problems with food (high prices, low availability, low access)   खाने की समस्या (ज्यादा कीमत/महंगा, कम उपलब्धता, कम सुलभता/पहुँच)   1. Shops being closed-essential items   दुकानों का बंद होना   1. Long distance travel restrictions   लम्बी दूरी की यात्रा पर प्रतिबन्ध   1. Not visiting family/friends (social distancing)   परिवार/ दोस्तों के पास न जाना (सामाजिक दूरी)   1. Staying indoors (quarantine/self-quarantine)   घर के भीतर रहना (क्वारंटाइन/ सेल्फ-क्वारंटाइन)   1. Household members have gotten sick   घर के सदस्य बीमार हो गए   1. Fear of household members getting sick   घर के सदस्यों के बीमार होने का डर   1. Less able to access health services   स्वास्थ्य सेवाओं की कम सुलभता   1. More household arguments   अधिक घरेलू बहस   1. Burden of additional household duties   अतिरिक्त घरेलू जिम्मेदारियों का बोझ   1. Burden of additional work   अतिरिक्त काम का बोझ   1. Travel long distances for work because of lack of transport   परिवहन की कमी की वजह से काम के लिए लम्बी दूरी की यात्रा करना   1. HH members who were visiting other places got stuck/couldn’t travel back home.   परिवार के जो सदस्य बाहर गए थे, वे वहीँ फंस गए ,वापस नहीं आ पाए  95. Other specify (______________) अन्य (बताएं ) |

# **Module G: Permission for repeat call [4 Questions]**

**मोड्यूल G : दोबारा कॉल करने की अनुमति**

| **Question** | **Response options** |
| --- | --- |
| 1. Can we call you again after a few days to speak with you again?   क्या हम आपसे दोबारा बात करने के लिए कुछ दिनों के बाद फिर से कॉल कर सकते हैं?  END SURVEY IF NO | 0. No नहीं  1. Yes हाँ |
| 1. Can we call on the same phone number?   क्या हम इसी फोन नंबर पर कॉल कर सकते है? | 0. No नहीं  1. Yes हाँ |
| 1. Do you have an alternate phone number?   क्या आपके पास वैकल्पिक/ अतिरिक्त फोन नंबर है? | 0. No नहीं  1. Yes हाँ |
| 1. *If Q3=1, then ask for alternate phone number*   *यदि Q3=1 हो तो वैकल्पिक/ अतिरिक्त फोन नंबर पूछें* | Phone number:  फोन नंबर |

**Thank you for giving us time to talk with you. हम से बात करने के लिए समय निकालने के लिए धन्यवाद**
